# Sequence features governing aggregation or degradation of prion-like proteins

**DOI:** 10.1371/journal.pgen.1007517

**Published:** 2018-07-13

**Authors:** Sean M. Cascarina, Kacy R. Paul, Satoshi Machihara, Eric D. Ross

**Affiliations:** Department of Biochemistry and Molecular Biology, Colorado State University, Fort Collins, Colorado, United States of America; University of Massachusetts Amherst, UNITED STATES

## Abstract

Enhanced protein aggregation and/or impaired clearance of aggregates can lead to neurodegenerative disorders such as Alzheimer’s Disease, Huntington’s Disease, and prion diseases. Therefore, many protein quality control factors specialize in recognizing and degrading aggregation-prone proteins. Prions, which generally result from self-propagating protein aggregates, must therefore evade or outcompete these quality control systems in order to form and propagate in a cellular context. We developed a genetic screen in yeast that allowed us to explore the sequence features that promote degradation versus aggregation of a model glutamine/asparagine (Q/N)-rich prion domain from the yeast prion protein, Sup35, and two model glycine (G)-rich prion-like domains from the human proteins hnRNPA1 and hnRNPA2. Unexpectedly, we found that aggregation propensity and degradation propensity could be uncoupled in multiple ways. First, only a subset of classically aggregation-promoting amino acids elicited a strong degradation response in the G-rich prion-like domains. Specifically, large aliphatic residues enhanced degradation of the prion-like domains, whereas aromatic residues promoted prion aggregation without enhancing degradation. Second, the degradation-promoting effect of aliphatic residues was suppressed in the context of the Q/N-rich prion domain, and instead led to a dose-dependent increase in the frequency of spontaneous prion formation. Degradation suppression correlated with Q/N content of the surrounding prion domain, potentially indicating an underappreciated activity for these residues in yeast prion domains. Collectively, these results provide key insights into how certain aggregation-prone proteins may evade protein quality control degradation systems.

## Author summary

Protein aggregation is associated with a variety of diseases, including Alzheimer’s disease and Amyotrophic Lateral Sclerosis. Cells possess a number of factors that can recognize aggregation-prone protein features and prevent aggregation. One common way this is achieved is through the pre-emptive degradation of aggregation-prone proteins. While considerable progress has been made in understanding how the amino acid sequence of a protein relates to intrinsic aggregation propensity, little is known about how aggregation-prone proteins avoid intracellular anti-aggregation systems. We used a genetic screen in yeast to define sequence features of aggregation-prone domains that lead to degradation or prion aggregation as it occurs in the context of eukaryotic protein quality control factors. Unexpectedly, we found that only a subset of aggregation-promoting amino acids could effectively stimulate degradation of an aggregation-prone domain. Furthermore, this degradation-promoting effect could be suppressed by classical prion domain features. Our results highlight the complex interplay between pre-emptive protein degradation and protein aggregation, and implicate the unusual composition of yeast prion domains in preventing their degradation.

## Introduction

Protein misfolding disorders involve the conversion of native proteins into non-native, deleterious forms. Some misfolded proteins form highly ordered amyloid aggregates, stabilized by intermolecular cross-β sheets. Once formed, these aggregates can convert remaining soluble proteins to the aggregated form via a templated misfolding mechanism [1]. Harmful aggregates must be prevented, sequestered, disassembled, or degraded by cells to prevent disruption of essential cellular functions. Enhanced protein aggregation or impaired clearance of aggregates can lead to neurodegenerative disorders such as Alzheimer’s Disease, Parkinson’s Disease, Amyotrophic Lateral Sclerosis (ALS), and Huntington’s Disease (for review, see [2–9]).

Prion diseases represent a unique sub-class of protein misfolding disorders in which protein aggregates are infectious. Prions can arise *de novo* through protein misfolding events that convert native proteins into the infectious form, or may be acquired through environmental encounter with the infectious form [10]. Although first described in mammals, a number of prion proteins were later found to occur in budding yeast [11, 12].

*Saccharomyces cerevisiae* has been used extensively as a model organism to study prions [11, 13]. Discovery and characterization of the first two yeast prion proteins, Ure2 and Sup35, revealed that both proteins contain remarkably glutamine/asparagine (Q/N) rich prion domains [12, 14, 15]. The prion domains also contain relatively few charged and hydrophobic residues. Scrambling experiments demonstrated that the ability of Ure2 and Sup35 to form prions is largely dependent on the amino acid composition of the prion domains, rather than the primary amino acid sequence [16, 17]. Methods for scanning the yeast proteome for additional proteins with similar compositional features resulted in successful identification of new yeast prions [18–20]. To date, nine yeast proteins have been demonstrated to form aggregation-mediated prions [12, 18, 21–27]. The majority of these proteins also contain prion domains with high Q/N content and low charged/hydrophobic content.

Examination of the human proteome with more sophisticated composition-based search algorithms revealed a number of human proteins with “prion-like domains” (PrLDs), defined as domains that compositionally resemble yeast prion domains [5, 28]. Many of the top candidates (including TDP-43 [29, 30], FUS [31, 32], EWSR1 [33, 34], TAF15 [34–36], hnRNPA1 [37], hnRNPA2B1 [37], and TIA1 [38]) have been implicated in protein misfolding disorders. In addition to containing PrLDs, aggregates formed by these proteins are thought to spread throughout an individual in an infectious prion-like manner along a neuroanatomical path that parallels the progression of pathological symptoms [3, 39]. Furthermore, the PrLDs from hnRNPA1 and hnRNPA2B1 are able to support prion activity when substituted in place of the portion of the Sup35 prion domain that is responsible for nucleating prion activity [37, 40].

Although composition-based algorithms have been reasonably effective at identifying candidate yeast prion proteins and potential disease-associated human PrLDs, these algorithms are less effective at predicting the aggregation propensity of these domains or the effects of mutations [41]. One limitation of these methods is that while they assess the frequency with which amino acids occur in prion domains, this frequency may not reflect the importance of each amino acid in prion formation. To address this knowledge gap, we previously used a quantitative mutagenesis method to score the prion propensity of each amino acid in the context of a Q/N-rich prion domain [42]. Interestingly, although the yeast prions tend to be strikingly Q/N-rich, both glutamine and asparagine were found to have neutral prion propensity scores [42, 43]. Instead, many of the non-aromatic hydrophobic amino acids (I, M, and V) and the aromatic amino acids (F, W, and Y) were observed to have strong prion-promoting effects, implicating these amino acids as key nucleators of prion aggregation [42, 44, 45]. We then used these prion-propensity scores to create PAPA, a prion prediction algorithm optimized for yeast prion domains [46, 47]. PAPA is reasonably effective at predicting the prion propensity of Q/N-rich domains, as well as the effects of mutations on prion activity [45, 47, 48].

However, although the composition of the human PrLDs resembles the composition of yeast prion domains, the human PrLDs tend to be less Q/N-rich and contain a higher percentage of serine and glycine (for review, see [41]). Therefore, it is likely that prediction methods developed for yeast prion domains may not be optimized for human PrLDs. To understand how amino acid context (i.e. the starting composition) of PrLDs affects amino acid prion propensities, we sought to determine the prion propensity of each amino acid in the context of two model glycine (G) rich human PrLDs from hnRNPA1 and A2. As in the context of Q/N-rich yeast prion domains, we found that aromatic amino acids were strongly prion-promoting in the context of these G-rich PrLDs. However, contrary to their effect in Q/N-rich yeast prion domains, the non-aromatic hydrophobic amino acids were not strongly prion-promoting; instead, they served as a signal for targeted degradation of the G-rich PrLDs. This suggests that aromatic amino acids may have the unique capacity to increase the aggregation propensity of prion or prion-like domains while avoiding efficient detection by protein degradation systems. Furthermore, Q/N residues strongly inhibited degradation of the G-rich PrLDs, suggesting that they may help prevent degradation of prion and prion-like domains. Indeed, many of the same sequences that led to degradation in the context of the G-rich PrLD had no effect on turnover of a Q/N-rich prion domain. These results broaden our understanding of the proteostatic regulation of aggregation-prone proteins, and shed light on the role of Q/N residues within prion domains.

## Results

### Non-aromatic hydrophobic residues promote degradation in human PrLDs but not in a yeast prion domain

The core PrLDs from the human RNA-binding proteins hnRNPA1 and hnRNPA2 were chosen as model substrates to examine the sequence requirements for aggregation within G-rich PrLDs. Both proteins contain a C-terminal G-rich PrLD. Mutations in these domains cause ALS and multisystem proteinopathy in humans, increase their aggregation propensity *in vitro*, and cause muscle degeneration when the proteins are expressed in *Drosophila* [37, 48, 49].

We previously used a yeast prion system to examine the effect of mutations on the aggregation propensity of the hnRNPA1 and A2 PrLDs [37]. The yeast prion protein Sup35 contains three functionally distinct domains: an N-terminal prion domain that is necessary and sufficient for formation of prion aggregates; a C-terminal functional domain, which is involved in translation termination; and a highly charged middle domain [15, 50, 51]. The first 40 amino acids of the prion domain, referred to as the nucleation domain (ND), are very Q/N-rich and are responsible for nucleating prion aggregates [40]. We therefore replaced the Sup35 ND with the core PrLD from hnRNPA1 and hnRNPA2 to test whether these PrLDs could support prion activity [37]. These fusion proteins allowed us to use well-established Sup35 prion detection assays to probe the relationship between amino acid sequence and aggregation activity for the hnRNPA1 and hnRNPA2 PrLDs.

Formation of [*PSI*^+^], the prion form of Sup35, can be assayed by monitoring nonsense suppression of the *ade2-1* allele in the presence of tRNA suppressor *SUP16* [52]. *ade2-1* mutants are unable to grow on medium lacking adenine (SC-ade), and grow red on medium containing limited adenine (YPD) due to accumulation of a pigment derived from the substrate of the Ade2 enzyme; [*PSI*^+^] formation results in a low level of read-through of the *ade2-1* premature stop codon, allowing for growth on SC-ade, and formation of white colonies on YPD. The fusion proteins showed a number of hallmarks of mutation-dependent prion activity [37, 48], including; 1) spontaneous formation of *ADE*^+^ colonies, and an increase in *ADE*^*+*^ colony formation upon PrLD overexpression; 2) curability of the *ADE*^*+*^ phenotype by 4mM GuHCl, a treatment that cures [*PSI*^+^] [53]; 3) transmission of the phenotype by cytoduction; and 4) the formation of microscopically-visible foci in *ADE*^*+*^ cells and the absence of foci in *ade*- cells. Furthermore, the *in vitro* amyloid propensity, the formation of visible foci, and the frequency of appearance of the *ADE*^*+*^ phenotype could be influenced in a predictable manner with rationally-designed mutations derived from an established prion propensity scale.

**Fig 1:**
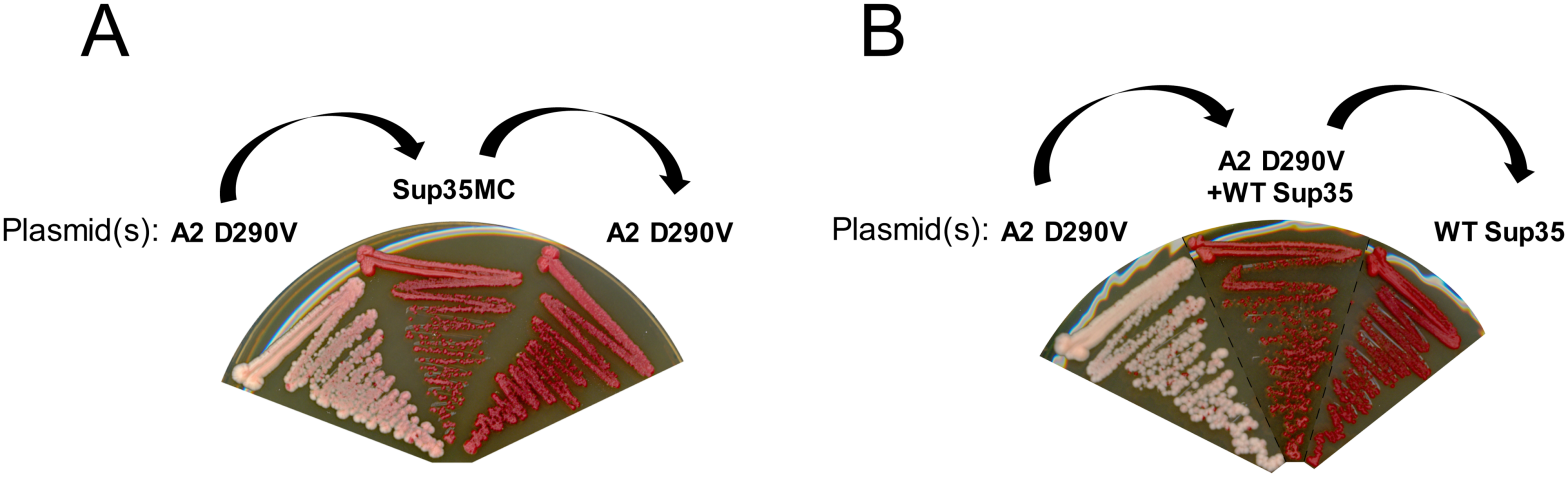
The A2-Sup35 protein exhibits PrLD-dependent prion activity. (A) A2-Sup35 prion maintenance requires continual expression of the A2-Sup35 prion domain. A covering plasmid expressing a copy of Sup35 lacking the prion domain was shuffled into a [*PRION*^+^] isolate (A2 D290V). In the [*PRION*^+^] strain, cells became *ade*^-^ upon loss of the A2-Sup35 plasmid, and remained *ade*^-^ when the plasmid was re-introduced. (B) A2-Sup35 prions are unable to convert wild-type Sup35. Co-expression of wild-type Sup35 in the [*PRION*^+^] strain resulted in a reversion to the *ade*^-^ phenotype that was maintained upon loss of the A2-Sup35 plasmid, suggesting that the A2-Sup35 prion form is not efficiently transmitted to wild-type Sup35.

These studies provide strong evidence that the fusion proteins form prions. However, some Sup35 mutants with modified prion domains can show similar nonsense suppression that is not due to Sup35 prion formation [54]. Therefore, we took additional steps to confirm the prion activity of the fusion proteins. Prion maintenance requires continuous expression of the prion protein. To provide additional evidence that the hnRNP-Sup35 fusions form canonical aggregation-mediated prions in yeast, we induced prion formation by overexpressing the A2 D290V PrLD in cells expressing hnRNPA2-Sup35(D290V) as the sole copy of Sup35 [37]. Two independent [*PRION*^+^] colonies were transformed with a Sup35 plasmid lacking the prion domain (Sup35MC) and passaged in the absence of selection for the A2-Sup35 plasmid. In both cases, the prions formed by the A2-Sup35 fusions were cured upon loss of the A2-Sup35 expressing vector, and only rarely spontaneously reappeared upon re-introduction of the vector, indicating that the prion phenotype required expression of the A2 PrLD to be maintained (Fig 1A). Since the hnRNP-Sup35 fusions contain a portion of the native Sup35 prion domain, we also examined whether the prions formed by the A2 PrLD were transferable to wild-type Sup35, which could suggest that the remainder of the Sup35 prion domain (rather than the A2 PrLD) was predominantly responsible for prion activity. Co-expression of wild-type Sup35 with A2 D290V suppressed the prion phenotype. After passaging these cells in the absence of selection for the A2-Sup35 plasmid, 23 out of 24 isolates that had not maintained the A2-Sup35 plasmid showed a [*prion*^-^] phenotype (Fig 1B), while the final isolate exhibited an atypical yellow phenotype with small colonies that resembled neither a [*PRION*^+^] or a [*prion*^-^] phenotype (potentially indicating contamination). This suggests that prions formed by the A2-Sup35 fusions were not sufficient to structurally convert Sup35 to a prion state. Collectively, these results indicate that the hnRNP-Sup35 fusions form canonical, PrLD-dependent prions.

**Fig 2:**
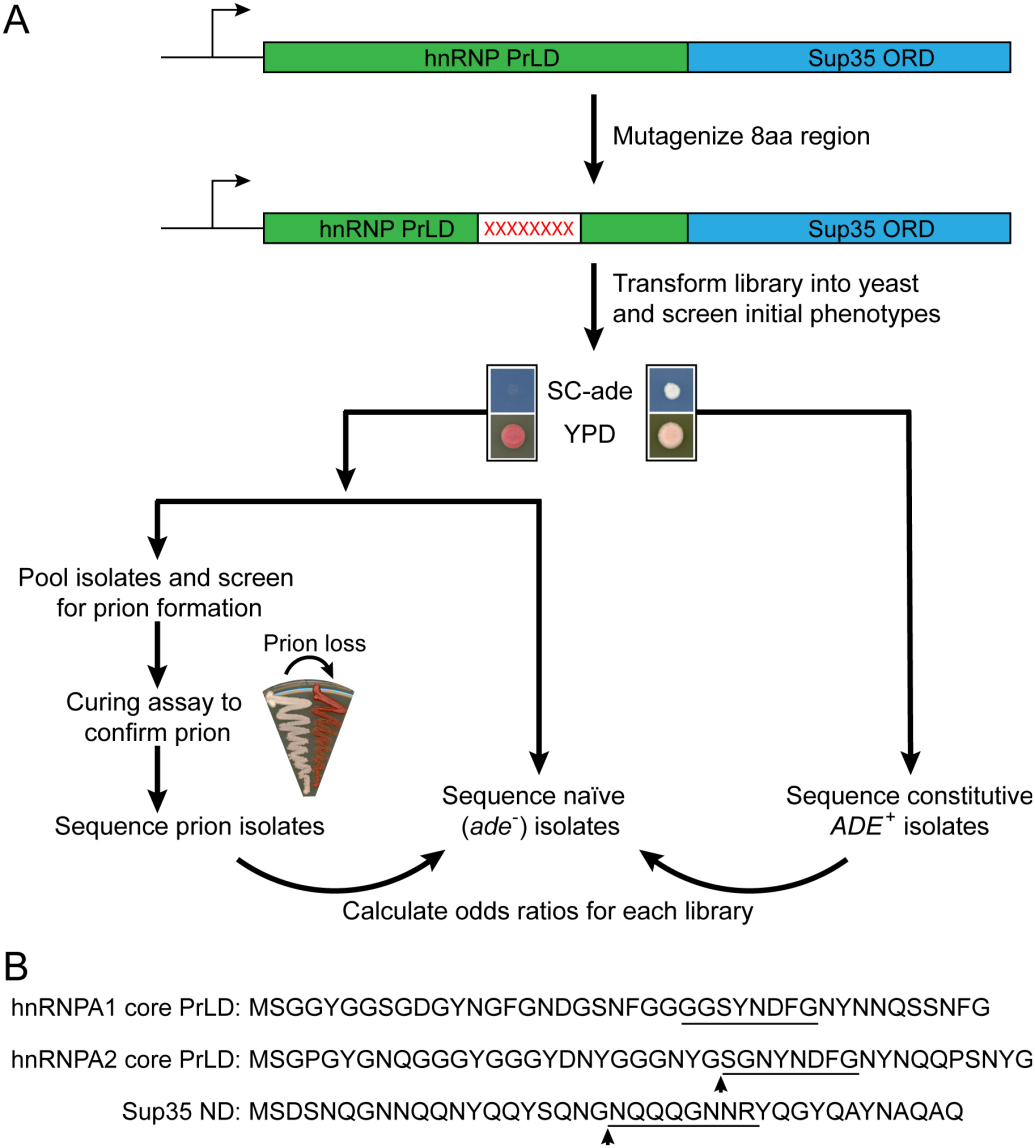
Mutagenesis method. (A) The Sup35 prion domain contains an N-terminal Q/N-rich prion nucleation domain, and an oligopeptide repeat domain. The nucleation domain in full-length Sup35 was replaced with the core PrLDs from hnRNPA1 and hnRNPA2. An 8-amino-acid segment in each of the fusion proteins was then randomly mutagenized. Mutants were expressed as the sole copy of Sup35 in the cell. Library members were screened for their initial adenine phenotype, and for the ability to form prions. (B) Sequences of the core PrLDs from hnRNPA1 and A2, and the nucleation domain of Sup35. The underlined segments of hnRNPA1 and A2 were mutagenized in this study, while the underlined segment of Sup35 was mutagenized previously [44]. Arrow heads indicate the sites of hydrophobic insertion in Figs 7A and 6B.

We previously developed a method to quantitatively score the effects of mutations on Sup35 prion activity [42, 44]. We replaced 8-amino acid segments of the prion domain with a random sequence, generating libraries of mutants. Each mutant was expressed as the sole copy of Sup35 in the cell. Randomly mutagenized libraries were plated onto medium lacking adenine to select for mutants that maintained the ability to form [*PSI*^+^]. This method was applied to various regions of wild-type and scrambled Sup35, including the Sup35 nucleation domain [42, 44]. Therefore, to examine how the sequence requirements for aggregation differ between Q/N-rich and G-rich PrLDs, we repeated this method, mutating the hnRNPA1-Sup35 and hnRNPA2-Sup35 fusions (herein referred to as A1-Sup35 and A2-Sup35 respectively; Fig 2A). As targets for mutagenesis, we selected segments with a mixture of predicted aggregation-promoting, aggregation-inhibiting, and neutral amino acids, near the site corresponding to a region previously mutagenized in Sup35 (Fig 2B).

Spontaneous [*PSI*^+^] formation is typically a stochastic and very rare event, occurring at a rate of less than 10^−6^ per generation [55]. By contrast, mutations that reduce Sup35 activity without causing prion aggregation will result in a constitutive *ADE*^*+*^ phenotype. Thus, to detect rare prion formation events from among a library of mutants, it is necessary to first eliminate mutants that have a constitutive *ADE*^*+*^ phenotype (Fig 2A). In previous screens with wild-type or scrambled Sup35, such constitutive *ADE*^*+*^ mutants were relatively rare, comprising ~5% of screened isolates [42, 44]. Unexpectedly, for the mutagenized A2-Sup35 and A1-Sup35 fusions, approximately 30–40% of the isolates were able to grow in the absence of adenine. These *ADE*^*+*^ isolates were not cured by treatment with 4mM GuHCl, suggesting that the growth on SC-ade resulted from non-prion-based inactivation of the hnRNP-Sup35 fusion proteins. As observed for [*PRION*^+^] isolates, replacement of the *A2-SUP35* plasmid with a plasmid expressing Sup35MC results in loss of the *ADE*^+^ phenotype. However, in contrast to [*PRION*^+^] strains, when plasmids expressing A1- or A2-Sup35 mutants were isolated from representative strains with a constitutive *ADE*^*+*^ phenotype and shuffled back into the parent strain, the *ADE*^*+*^ phenotype spontaneously re-appeared (S1 Fig). This indicates that the phenotype results from loss of activity of the A2-Sup35 fusion protein, not from mutations in other cellular proteins or from classical PrLD-dependent prion propagation. Finally, while prion formation was associated with increased levels of insoluble A2-Sup35 protein, the constitutive *ADE*^*+*^ mutants did not contain substantial amounts of insoluble A2-Sup35 protein (S2 Fig).

**Fig 3:**
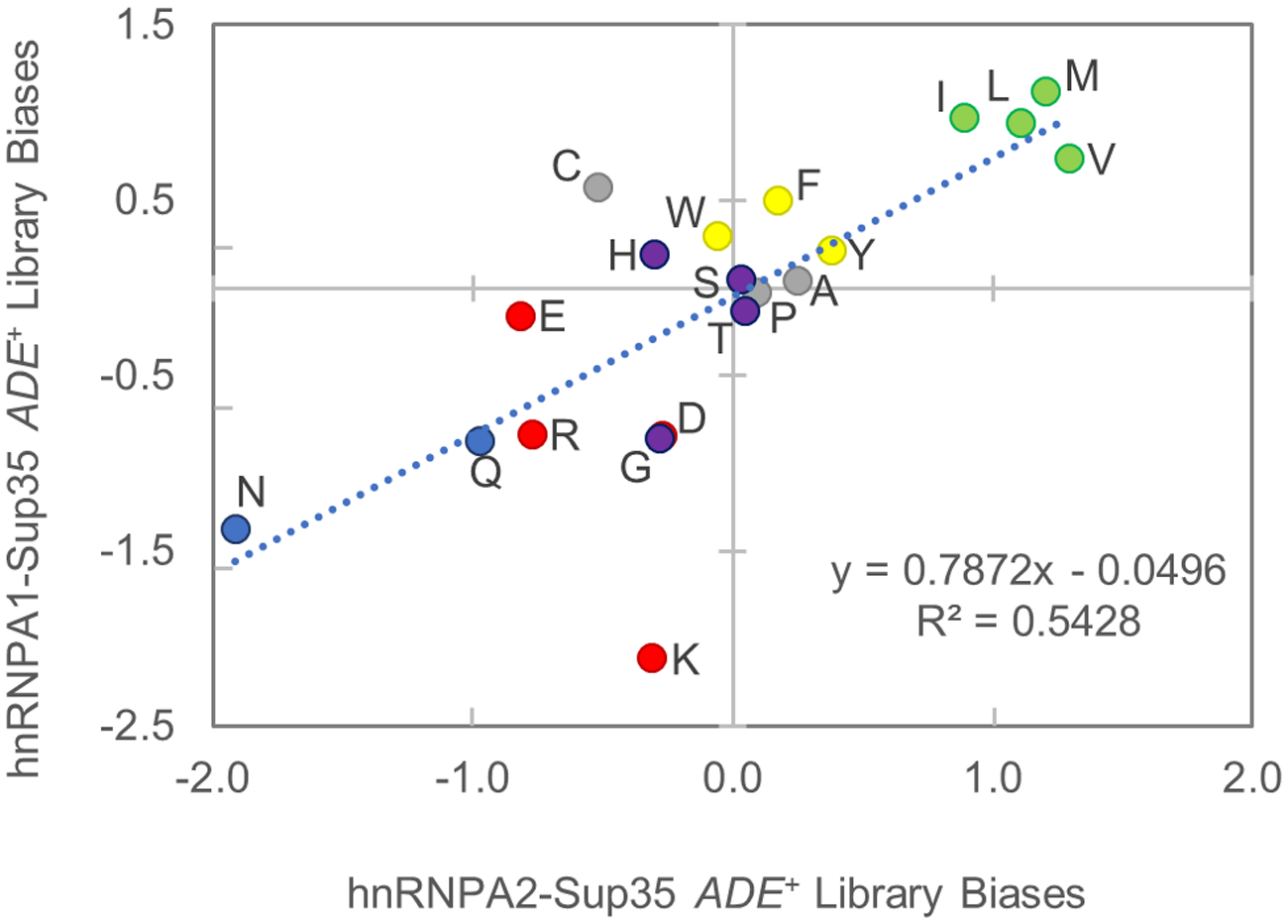
Similar amino acid biases govern the *ADE*^*+*^ phenotype within the A1 and A2 PrLD’s. Comparison of the log-odds ratio for each amino acid between the A1 and A2 libraries. The log-odds ratios reflects the degree of over-/under-representation of each amino acid among constitutive *ADE*^*+*^ isolates, relative to *ade*^*-*^ isolates. Colors correspond to the amino acid groups in Table 1.

Therefore, we sought to determine the basis of Sup35 inactivation among these isolates. We sequenced the mutagenized region of the *A1/A2-SUP35* gene from randomly selected *ade*^*-*^ and *ADE*^*+*^ isolates to determine whether specific sequence features were correlated with the *ADE*^*+*^ phenotype. For each amino acid, an odds ratio was calculated (Eq 1), representing the degree of over- or under-representation of the amino acid among *ADE*^*+*^ isolates (Table 1). For both libraries, each of the non-aromatic hydrophobic amino acids (I, L, M, and V) were over-represented among *ADE*^*+*^ isolates, while glutamine, asparagine, and each of the charged amino acids (D, E, K, and R) were under-represented (Table 1; Fig 3). Individually, not all of these biases reached the standard threshold of statistical significance (p < 0.05; Table 1). Grouping amino acids of similar physical properties can increase statistical significance by effectively increasing sample sizes. When considered as a group, the biases for hydrophobic amino acids, against charged amino acids, and against Q/N were each statistically significant in both libraries (P<0.01 in all cases; Table 1).

**Table 1:** Amino acid representation among *ADE*^+^ and *ade*^-^ isolates.

Amino Acids	hnRNPA2-Sup35 Fusion Library				hnRNPA1-Sup35 Fusion Library			
	Frequency		ln(OR_A_) ^ǂ^	*P value*	Frequency		ln(OR_A_) ^ǂ^	*P value*
	*ADE*^+^ Library	*ade*^-^ Library			*ADE*^+^ Library	*ade*^-^ Library		
Valine	0.078	0.023	1.30	7.8 x 10^−4^	0.083	0.042	0.74	0.049
Methionine	0.041	0.013	1.21	0.024	0.033	0.011	1.12	0.074
Leucine	0.125	0.045	1.11	1.7 x 10^−4^	0.100	0.042	0.94	6.3 x 10^−3^
Isoleucine	0.047	0.020	0.89	0.049	0.083	0.033	0.97	9.2 x 10^−3^
Tyrosine	0.057	0.040	0.38	0.29	0.038	0.031	0.21	0.65
Alanine	0.051	0.040	0.25	0.58	0.038	0.036	0.039	1.00
Phenylalanine	0.047	0.040	0.18	0.71	0.067	0.042	0.50	0.19
Proline	0.057	0.053	0.10	0.87	0.063	0.064	-0.023	1.00
Threonine	0.057	0.055	0.046	1.00	0.054	0.061	-0.13	0.86
Serine	0.128	0.125	0.031	0.91	0.125	0.119	0.052	0.90
Tryptophan	0.024	0.025	-0.057	1.00	0.033	0.025	0.30	0.62
Aspartic Acid	0.041	0.053	-0.27	0.59	0.021	0.047	-0.85	0.12
Glycine	0.068	0.088	-0.28	0.39	0.033	0.075	-0.85	0.034
Histidine	0.034	0.045	-0.30	0.56	0.050	0.042	0.19	0.69
Lysine	0.020	0.028	-0.31	0.63	0.004	0.033	-2.11	0.019
Cysteine	0.030	0.050	-0.52	0.25	0.063	0.036	0.58	0.17
Arginine	0.054	0.110	-0.77	9.4 x 10^−3^	0.058	0.125	-0.84	7.6 x 10^−3^
Glutamic Acid	0.014	0.030	-0.81	0.20	0.017	0.019	-0.16	1.00
Glutamine	0.014	0.035	-0.97	0.093	0.017	0.039	-0.87	0.15
Asparagine	0.014	0.085	-1.91	1.5 x 10^−5^	0.021	0.078	-1.38	2.9 x 10^−3^
*Groups of amino acids*								
Aromatic (FWY)	0.128	0.105	0.23	0.34	0.138	0.097	0.39	0.15
Charged (DEKR)	0.128	0.220	-0.65	2.0 x 10^−3^	0.100	0.225	-0.96	6.9 x 10^−5^
Hydrophobic (ILMV)	0.291	0.100	1.30	1.7 x 10^−10^	0.300	0.128	1.07	3.5 x 10^−7^
Polar (GHST)	0.287	0.313	-0.12	0.50	0.263	0.297	-0.17	0.41
QN	0.027	0.120	-1.59	3.8 x 10^−6^	0.038	0.117	-1.22	5.1 x 10^−4^

^*ǂ*^ Odds ratios (OR_*A*_) were calculated as in Eq 1, and represent the degree of overrepresentation or underrepresentation of each amino acid among constitutive *ADE*^+^ isolates.

**Fig 4:**
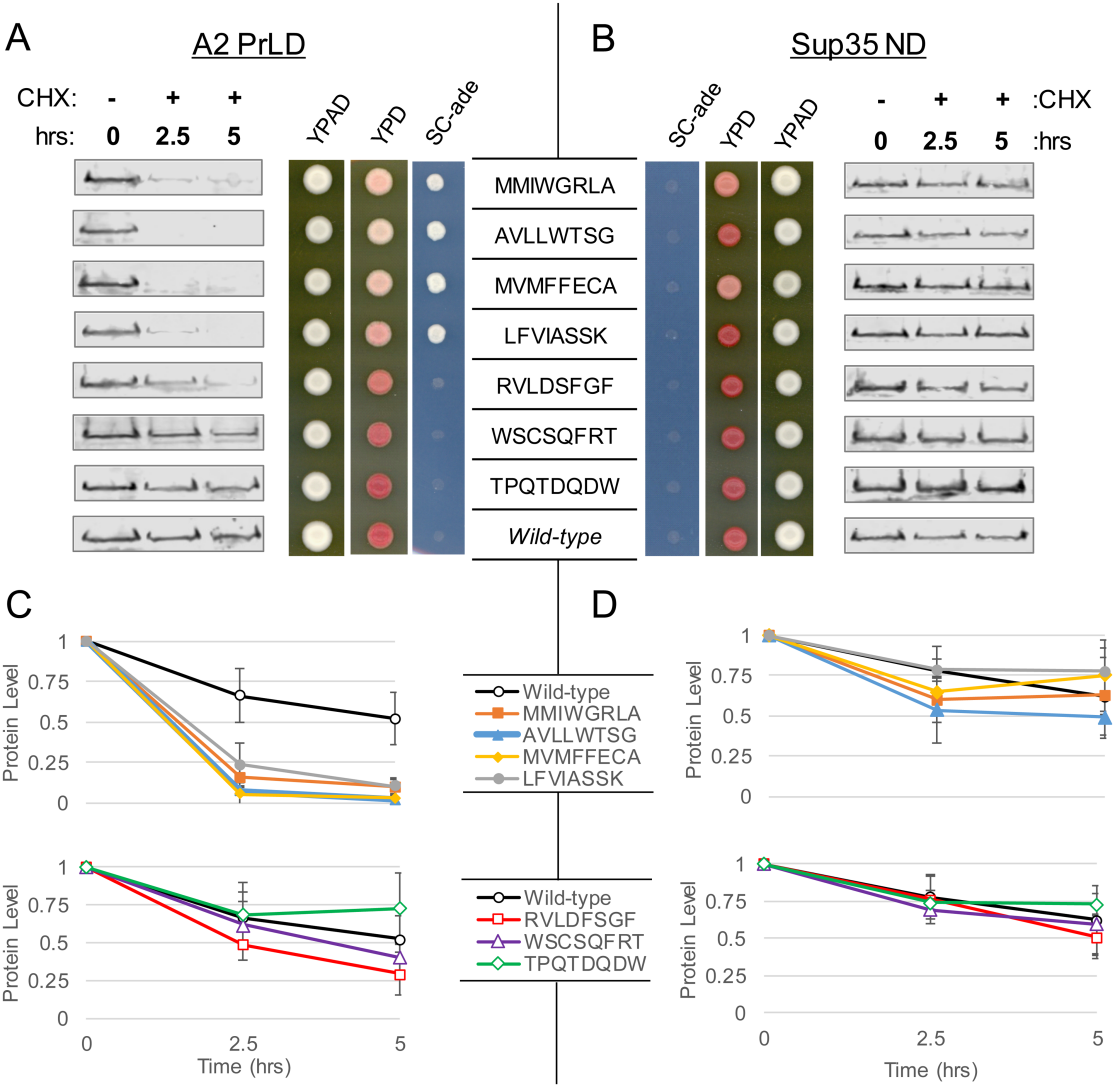
Hydrophobic peptides promote degradation of the A2 PrLD but not the Sup35 nucleation domain. (A) The *ADE*^*+*^ phenotype for A2-Sup35 mutants is associated with increased protein turnover. *ADE*^*+*^ and *ade*^*-*^ isolates expressing the indicated A2-Sup35 fusion as the sole copy of Sup35 in the cell were plated on SC-ade and YPD to confirm phenotypes originally observed in the mutagenesis/screening method. Protein levels were assessed by western blot after treatment with CHX. The *ADE*^*+*^ phenotype is associated with accelerated degradation of the fusion protein. Wild-type sequences are the respective sequences from wild-type Sup35 and the A2 PrLD. (B) In the context of the Sup35 ND, all peptides conferred an *ade*^*-*^ phenotype and did not accelerate degradation. Western blots were quantified for all A2 PrLD (C) and Sup35 ND (D) mutants. Data represent means ± SDs (n≥3).

One possible explanation for the *ADE*^*+*^ phenotype is that the hnRNP-Sup35 fusions could be poorly expressed or rapidly degraded, causing a decrease in steady state levels of the fusion proteins. To test this possibility, four representative A2-Sup35 isolates that exemplified the amino acid biases among the *ADE*^*+*^ library were selected for comparison with randomly selected isolates from the *ade*^*-*^ library. The *ADE*^*+*^ and *ade*^*-*^ phenotypes originally observed for these isolates were confirmed by spotting onto SC-ade, YPD, and YPAD (Fig 4A). Previous studies suggest that an *ADE*^*+*^ phenotype is observed when steady-state Sup35 levels drop below about 40% of wild-type [56]. In synthetic complete medium, all four *ADE*^*+*^ isolates had steady-state A2-Sup35 levels that were less than 40% of wild-type, while three of four isolates from the *ade*^*-*^ library had steady state A2-Sup35 levels above this threshold (S3A Fig). When cells were shifted to medium lacking adenine, A2-Sup35 levels dropped for all eight strains, but showed lowest levels for the four *ADE*^*+*^ isolates. Furthermore, as a group, steady state protein levels for *ADE*^+^ isolates were significantly lower (p < 0.001) than the grouped protein levels for *ade*^-^ isolates in both synthetic complete and adenine-deficient synthetic complete media.

Exposed hydrophobic patches are known in some cases to trigger protein degradation [57, 58]. Therefore, we hypothesized that the lower average expression levels seen among the *ADE*^*+*^ isolates might be due to increased degradation. Cycloheximide (CHX) globally inhibits translation by preventing translocation of the ribosome along mRNA, providing a convenient tool to assay protein turnover [59]. After treatment with CHX, the fusion proteins within *ADE*^*+*^ isolates were rapidly degraded (Fig 4A). Three of the four *ADE*^*+*^ isolates contained little or no detectable A2-Sup35 by 2.5 hours after addition of CHX, while the fourth showed a substantial decrease in A2-Sup35 levels over the 5 hour timecourse (Fig 4A). By contrast, A2-Sup35 levels remained relatively stable or decreased only slightly over a period of 5 hours after addition of CHX for all of the *ade*^*-*^ isolates, as well as for the wild-type A2-Sup35 fusion (Fig 4A). These results suggest that hydrophobic amino acids trigger degradation of the A2-Sup35 fusions.

Interestingly, random mutagenesis of the Sup35 prion domain yielded very few isolates with the degradation phenotype in the initial screen [44], suggesting that the Sup35 prion domain can buffer the effects of degradation-promoting peptides. Indeed, when the degradation-promoting 8-amino acid sequences from the A2-Sup35 library were substituted into the corresponding region of the Sup35 prion domain, each of the proteins resulted in phenotypically *ade*^*-*^ cells (Fig 4B), and maintained steady-state Sup35 levels well above the 40% of wild-type (S3B Fig). Furthermore, none of the peptides accelerated the degradation rate of Sup35 over 5 hours (Fig 4B). Therefore, while the A2 PrLD is susceptible to the degradation-promoting effects of hydrophobic amino acids, the Sup35 prion domain can mask these effects and resist degradation.

### Degradation of the A2 PrLD is proteasome-dependent

**Fig 5:**
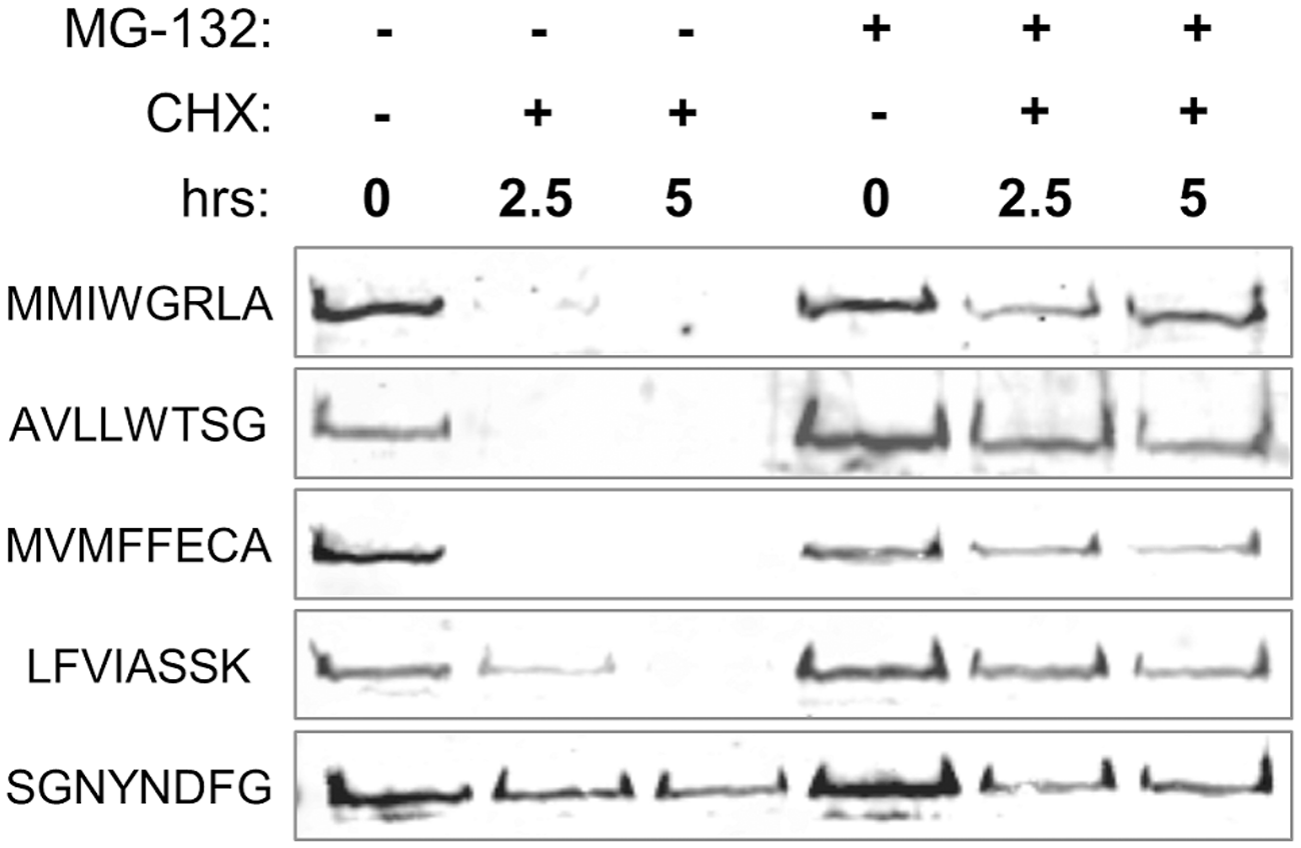
Degradation of A2 PrLDs occurs via the ubiquitin-proteasome system. Addition of MG-132 (+) 1hr prior to the addition of CHX prevents degradation of A2-Sup35 fusions (SGNYNDFG is the sequence in the corresponding region of the wild-type A2 PrLD).

The ubiquitin-proteasome system is one of the main protein recycling pathways in eukaryotic cells. MG-132, a commonly used proteasome inhibitor, is effective in yeast lacking the pleiotropic drug resistance 5 gene (*pdr5Δ*). To assess whether degradation of the A2-Sup35 proteins occurs via the proteasome, *PDR5* was deleted from the genome, and the turnover of the A2-Sup35 proteins was assessed in the presence or absence of MG-132. Pre-treatment with MG-132 for 1 hour prior to addition of CHX resulted in nearly complete stabilization of the degradation-prone A2-Sup35 fusions over the 5 hour timecourse (Fig 5). This result suggests that the *ADE*^*+*^ phenotype is due to enhanced turnover of the A2-Sup35 fusion proteins via the ubiquitin-proteasome system.

### Degradation-prone sequences can be predicted by amino acid composition

Since degradation-promoting sequences failed to cause degradation of Sup35 (Fig 4B), we reasoned that our previous dataset from random mutagenesis of Sup35 [44] would contain some peptide sequences that did not cause degradation in the context of the Sup35 prion domain, but would promote degradation of the A2-Sup35 fusion protein. To identify potential degradation-promoting sequences, each peptide from the library was scored by summing the log-odds ratios from Table 1 for the eight amino acids in the mutagenized region. Three sequences predicted to promote degradation (i.e., sequences enriched in non-aromatic hydrophobic residues, with few charged or Q/N residues) were selected from the dataset.

**Fig 6:**
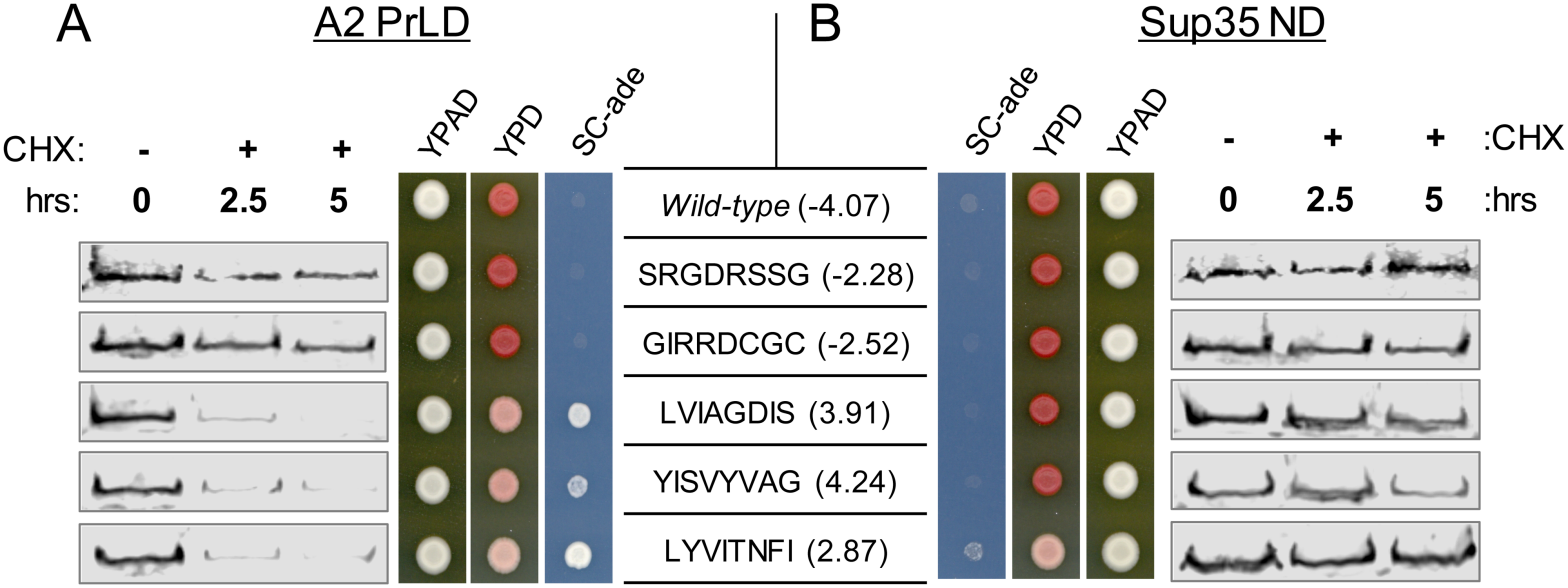
Amino acid degradation scores are sufficient to identify degradation-promoting and inhibiting peptides from an independent peptide library. (A) Predicted degradation-promoting (LVIAGDIS, YISVYVAG, and LYVITNFI) or inhibiting (SRGDRSSG and GIRRDCGC) peptides were substituted into the A2 PrLD (numbers in parentheses indicate the sum of the individual amino acid scores derived from the A2 PrLD degradation library). Predicted degradation-promoting peptides led to an *ADE*^*+*^ phenotype and accelerated degradation of the A2 PrLD. Predicted degradation-inhibiting peptides led to the *ade*^*-*^ and showed no increase in degradation rate of the A2-Sup35 fusion. (B) In the context of the Sup35 ND, all peptides were stable over 5 hrs and conferred a predominantly *ade*^*-*^ phenotype.

When substituted into A2-Sup35, all three predicted degradation-promoting peptides led to enhanced turnover of A2-Sup35 and characteristic degradation phenotypes, albeit to varying degrees (Fig 6A). All three strains appeared light pink on YPD, and growth on SC-ade correlated qualitatively with the degree of degradation conferred by each peptide. Additionally, two sequences predicted to have no effect on A2-Sup35 turnover (i.e., sequences enriched in charged and polar residues) were chosen from the same dataset as controls. When substituted into A2-Sup35, neither peptide enhanced degradation, and both strains displayed the associated *ade*^*-*^ phenotypes (Fig 6A). By contrast, four of the five peptides substituted into the Sup35 prion domain had little effect on turnover and resulted in the characteristic *ade*^*-*^ phenotype (Fig 6B), while the fifth showed modest degradation and only a weak *ADE*^*+*^ phenotype. These results demonstrate that the compositional biases originally observed in the *ADE*^+^ libraries are sufficient to predictively categorize sequences as degradation-promoting or degradation-inhibiting.

### Hydrophobic residues induce degradation or prion formation at a similar threshold

**Fig 7:**
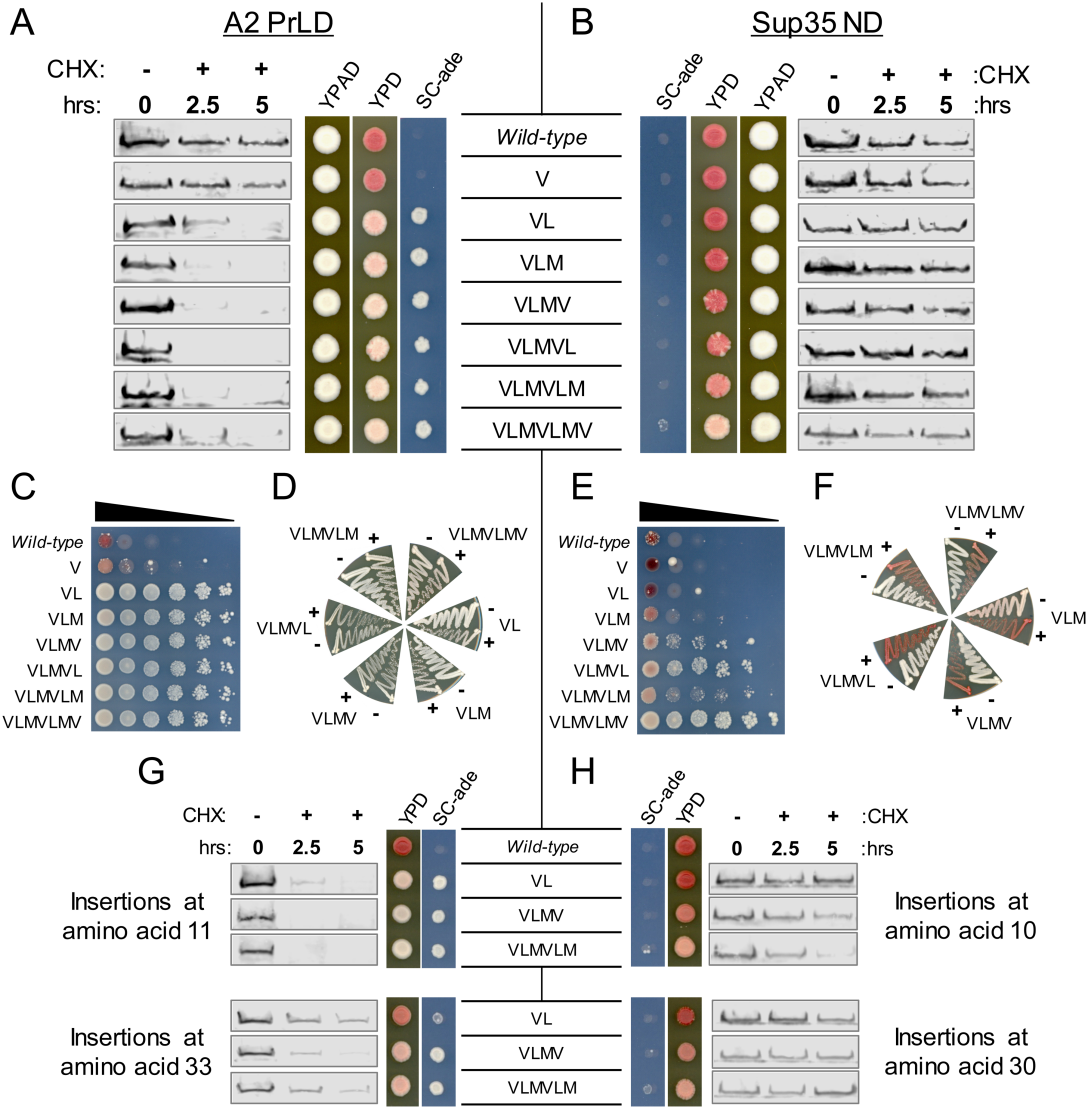
Degradation of the A2 PrLD and prion aggregation of Sup35 occur at similar hydrophobic content thresholds. (A) Two or more hydrophobic residues inserted into the A2 PrLD resulted in a robust *ADE*^*+*^ phenotype and accelerated degradation of the A2 PrLD. (B) Insertion of three or more hydrophobic residues into Sup35 led to a progressive increase in the frequency of white sectors on YPD without affecting Sup35 turnover. (C) To quantify the frequency of *ADE*^*+*^ colony formation, serial dilutions of cells expressing each A2-Sup35 fusion were plated onto SC-ade. Degradation of the A2 PrLD upon insertion of two or more hydrophobic residues was correlated with a binary-like switch from *ade*^-^ to *ADE*^+^. (D) *ADE*^+^ isolates from the A2 PrLD mutants were not curable by GuHCl. To test for curability of the *ADE*^+^ phenotype, individual *ADE*^+^ colonies were streaked on YPD (-) or YPD plus 4mM GuHCl (+), and then re-streaked onto YPD to test for loss of the *ADE*^+^ phenotype. (E) Insertion of three or more hydrophobic residues in the Sup35 ND leads to a progressive increase in *ADE*^+^ growth. (F) *ADE*^+^ isolates from the Sup35 mutants were curable by GuHCl, consistent with the *ADE*^*+*^ phenotype resulting from prion formation. (G) Insertion of hydrophobic amino acids at other positions in the A2 PrLD also promoted protein degradation. (H) Insertion of hydrophobic amino acids at other positions in the Sup35 nucleation domain had little or no effect on protein turnover.

The sequences obtained through random mutagenesis are heterogeneous with respect to composition and sequence. To more rigorously define the minimum number of non-aromatic hydrophobic residues required to accelerate the rate of degradation or prion formation, hydrophobic content was progressively increased in WT A2-Sup35 and WT Sup35. Valine, leucine, and methionine (the hydrophobic residues most over-represented in the *A2-Sup35 ADE*^*+*^ library) were inserted in an alternating fashion adjacent to the region targeted for random mutagenesis (Figs 2B and 7).

As few as two hydrophobic residues were sufficient to slightly increase turnover of A2-Sup35, as indicated by western blot and the characteristic *ADE*^*+*^ phenotype (Fig 7A). Three hydrophobic residues further accelerate A2-Sup35 degradation, and four to seven hydrophobic residues caused almost complete loss of A2-Sup35 by 2.5 hours after the addition of CHX. Two or fewer hydrophobic residues inserted into Sup35 resulted in uniform *ade*^*-*^ phenotypes, whereas three or more hydrophobic residues resulted in the appearance of white sectors, which are classical indications of prion formation (Fig 7B). Strikingly, the degree of sectoring increased in a dose-dependent fashion as hydrophobic content increased. Elimination of the [*PIN*^+^] prion did not affect the degradation of A2-Sup35 or stability of Sup35 upon insertion of hydrophobic residues (S4 Fig).

To more accurately quantify the frequency of *ADE*^*+*^ colony formation by each mutant, serial dilution of each mutant was plated on SC-ade, starting from a higher density than originally assayed. Fewer than two hydrophobic residues in A2-Sup35 resulted in minor growth only at high cell density, whereas two or more hydrophobic residues resulted in robust growth even at very low cell density (Fig 7C). Treatment with GuHCl did not alter the color phenotype on YPD (Fig 7D), suggesting that the *ADE*^*+*^ growth was not due to prion formation.

By contrast, three or more hydrophobic residues in Sup35 resulted in a progressive increase in the frequency of *ADE*^*+*^ colonies, consistent with the progressive increase in sectoring observed on YPD for these mutants (Fig 7E). Treating the cells with GuHCl reverted the *ADE*^*+*^ phenotype to an *ade*^*-*^ phenotype (Fig 7F), confirming that growth on SC-ade was due to the formation of *bona fide* prions.

These results were not unique to these specific positions within Sup35 and A2-Sup35. We made additional hydrophobic insertions one-quarter and three-quarters of the way through the Sup35 ND and the hnRNPA2 PrLD (positions 10 and 30 for Sup35; positions 11 and 33 for A2; Fig 2B). As with the original hydrophobic insertions (Fig 7A), insertions at both additional positions in the A2 PrLD resulted in increased degradation, although the effects of insertion were weaker at position 33 (Fig 7G). Likewise, Sup35 was far more resistant to the degradation-promoting effects of hydrophobic amino acids at both positions, although modest degradation was observed when six hydrophobic amino acids were inserted at position 10 (Fig 7H).

**Fig 8:**
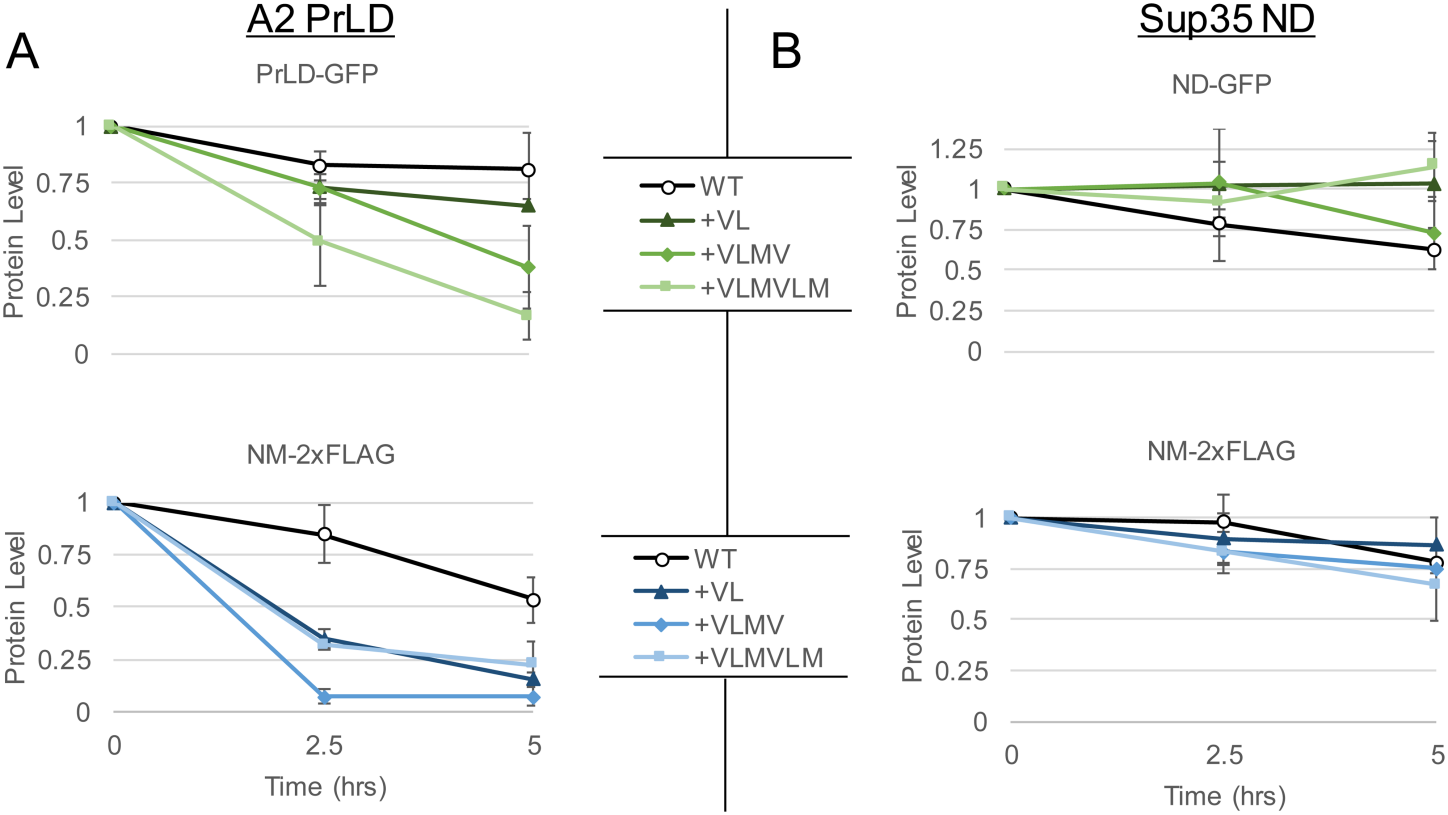
Degradation of the A2 PrLD and stability of the Sup35 ND upon insertion of hydrophobic residues do not depend on the Sup35 oligopeptide repeat domain (ORD), M-domain, or C-domain. Progressively increasing hydrophobic content in the A2 PrLD (A) when fused to GFP alone (*top*) or with the remainder of the Sup35NM domains and tandem FLAG tags (*bottom*) enhances degradation rate of the A2 PrLD fusion proteins. By contrast, insertion of hydrophobic residues in the Sup35 ND (B) fused to GFP (*top*) or Sup35NM-2xFLAG (*bottom*) does not decrease stability of the Sup35 ND fusion proteins. Data represent means ± SDs (n = 3).

It is possible that physical interactions between the Sup35ND and the remainder of the Sup35 sequence or with native Sup35 binding partners are responsible for the apparent stability of the Sup35 ND. However, insertion of hydrophobic residues in the A2 PrLD alone fused to GFP resulted in a progressive increase in degradation rate (Fig 8A, *top*), whereas insertion of hydrophobic residues in the Sup35 ND had no effect on degradation (Fig 8B, *top*). Nearly identical trends were observed for FLAG-tagged version of the A2-Sup35 and Sup35 NM domains (Fig 8, *bottom*).

Collectively, these results demonstrate that the Sup35 ND can mask the degradation-promoting effects of hydrophobic residues, and that this effect is not dependent on the remainder of the protein.

### Aromatic amino acids increase prion propensity in the human PrLDs without promoting protein turnover

The features promoting degradation of the A2 PrLD are consistent with previous studies indicating that the degradation machinery recognizes exposed hydrophobic segments, and that there is a strong correlation between the sequence features that promote aggregation and degradation [57, 58]. We were interested in whether this correlation is absolute, or whether there are sequence features that can promote aggregation of the G-rich PrLDs without promoting degradation. Our A1- and A2-Sup35 fusions provide a useful system for comparing the sequence requirements for degradation versus aggregation. To determine whether specific sequence features could promote prion aggregation without triggering degradation, isolates with an initial *ade*^-^ phenotype were plated onto medium lacking adenine to screen for the ability to spontaneously form prions (Fig 2A). [*PRION*^*+*^] isolates were confirmed by curing with GuHCl, and the mutagenized *A1/A2-SUP35* gene in each was sequenced. Sequences from each library were pooled, and the prion propensity scores for each amino acid were determined, as described previously ([42, 44]; Eq 4).

Interestingly, while both non-aromatic and aromatic hydrophobic residues were strongly prion-promoting within the Q/N-rich Sup35 ND [42, 44], only aromatic amino acids were significantly over-represented among [*PRION*^*+*^] isolates for the A2-Sup35 and A1-Sup35 libraries; non-aromatic hydrophobic residues were approximately equally represented among [*PRION*^*+*^] and *ade*^*-*^ isolates (Table 2). Furthermore, Q/N residues were significantly under-represented among A2-Sup35 [*PRION*^*+*^] isolates, although their effects were mixed among A1-Sup35 [*PRION*^*+*^] isolates. Together, these results suggest that a hitherto unappreciated property of aromatic amino acids is the unique ability to promote protein aggregation of prion and prion-like domains, while avoiding detection by the degradation machinery.

**Table 2:** Amino acid representation among [*PRION*^+^] and *ade*^-^ isolates.

Amino Acids	hnRNPA2-Sup35 Fusion Library				hnRNPA1-Sup35 Fusion Library			
	Frequency		ln(OR_*P*_) ^ǂ^	*P value*	Frequency		ln(OR_*P*_) ^ǂ^	*P value*
	[*PRION*^+^] Library	*ade*^-^ Library			[*PRION*^+^] Library	*ade*^-^ Library		
Phenylalanine	0.093	0.040	0.90	0.011	0.069	0.042	0.53	0.13
Tyrosine	0.088	0.040	0.84	0.018	0.069	0.031	0.86	0.028
Tryptophan	0.037	0.025	0.41	0.45	0.020	0.025	-0.24	0.80
Threonine	0.079	0.055	0.38	0.30	0.049	0.061	-0.23	0.61
Valine	0.032	0.023	0.38	0.44	0.053	0.042	0.25	0.58
Isoleucine	0.028	0.020	0.34	0.58	0.026	0.033	-0.24	0.65
Alanine	0.051	0.040	0.25	0.54	0.036	0.036	0.0021	1.00
Proline	0.060	0.053	0.14	0.71	0.026	0.064	-0.93	0.026
Methionine	0.014	0.013	0.11	1.00	0.030	0.011	1.00	0.10
Histidine	0.046	0.045	0.030	1.00	0.056	0.042	0.31	0.47
Glycine	0.083	0.088	-0.053	1.00	0.049	0.075	-0.45	0.20
Glutamic Acid	0.028	0.030	-0.079	1.00	0.007	0.019	-1.10	0.19
Leucine	0.042	0.045	-0.080	1.00	0.053	0.042	0.25	0.58
Arginine	0.093	0.110	-0.19	0.58	0.092	0.125	-0.34	0.21
Serine	0.093	0.125	-0.34	0.29	0.145	0.119	0.22	0.36
Glutamine	0.023	0.035	-0.43	0.47	0.023	0.039	-0.54	0.27
Cysteine	0.032	0.050	-0.45	0.41	0.039	0.036	0.093	0.84
Aspartic Acid	0.032	0.053	-0.50	0.31	0.033	0.047	-0.38	0.43
Asparagine	0.037	0.085	-0.88	0.029	0.109	0.078	0.37	0.18
Lysine	0.009	0.028	-1.11	0.15	0.016	0.033	-0.72	0.22
*Groups of amino acids*								
Aromatic (FWY)	0.218	0.105	0.86	2.7 x 10^−4^	0.158	0.097	0.55	0.025
Charged (DEKR)	0.162	0.220	-0.38	0.092	0.148	0.225	-0.51	0.013
Hydrophobic (ILMV)	0.116	0.100	0.16	0.58	0.161	0.128	0.27	0.22
Polar (GHST)	0.301	0.313	-0.054	0.78	0.299	0.297	0.010	1.00
Q/N	0.060	0.120	-0.76	0.017	0.132	0.117	0.14	0.64

^*ǂ*^ Odds ratios (OR_*p*_) were calculated as in Eq 3, and represent the degree of overrepresentation or underrepresentation of each amino acid among prion isolates.

**Fig 9:**
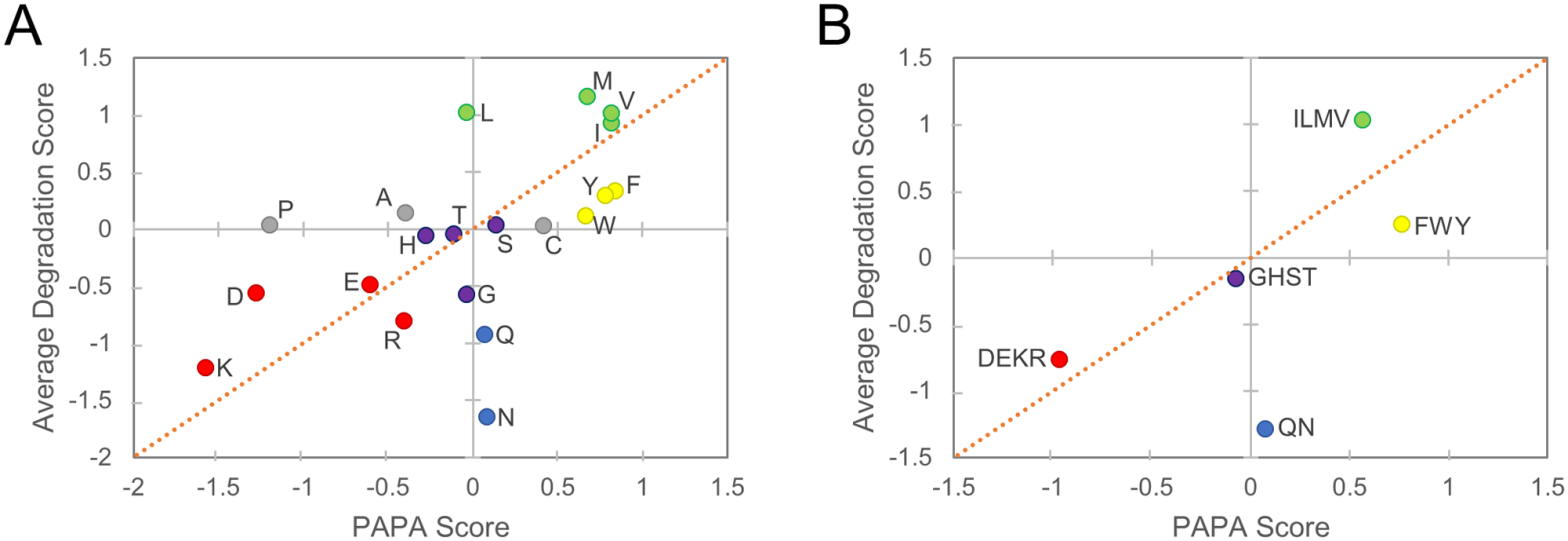
Yeast prion domains are enriched in amino acids that are prion-prone but not degradation-promoting. Average degradation scores from the A1 PrLD and A2 PrLD libraries are plotted against yeast prion propensity scores for individual amino acids (A) or amino acid groups (B). Within native yeast prion domains, commonly occurring amino acids (Q, N, and aromatic amino acids) exhibit a combination of high prion propensity and low degradation propensity. Colors correspond to the amino acid groups in Table 1 and Fig 3.

Indeed, while there is a statistically significant (P = 0.008 by Spearman rank analysis) correlation between the prion propensity (as scored by PAPA) of each amino acid and its propensity to promote degradation (Fig 9), there are five amino acids which have substantially lower degradation propensities than would be predicted by their prion propensities: the three aromatic amino acids, glutamine, and asparagine. Strikingly, these amino acids are all overrepresented among yeast prion proteins. While both aromatic and non-aromatic hydrophobic amino acids strongly promote prion formation [42], candidate prion domains with prion activity tend to contain more aromatic residues and fewer aliphatic residues than candidate prion domains with no detectable prion activity [44]. Likewise, although serine, glycine, threonine, glutamine, and asparagine each promote intrinsic disorder and have similar prion propensities [42], Q/N residues are far more common among yeast prion domains.

Collectively, these results suggest a possible explanation for the amino acid biases observed among yeast prion domains. Many components of protein quality control systems act specifically to antagonize protein aggregation. Therefore, proteins that form observable protein aggregates must possess mechanisms to avoid or outcompete antagonistic proteostasis machinery. Yeast prion domains tend to favor amino acids that promote aggregation while being poorly recognized by the degradation machinery.

### Q/N residues stabilize Sup35

**Fig 10:**
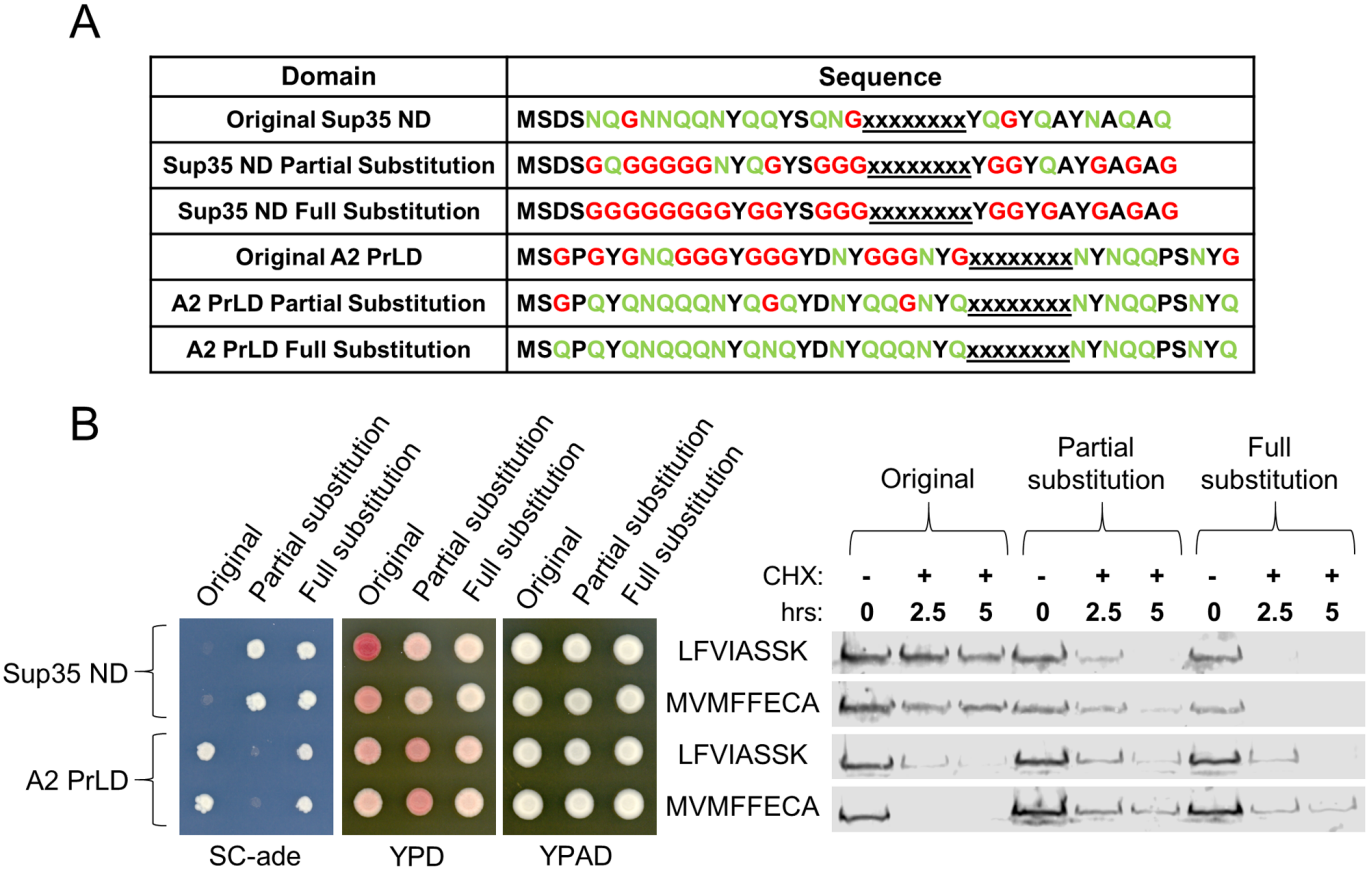
High Q/N content of Sup35 antagonizes degradation. (A) Sequences of the partial or full Q/N/G substitution constructs. Glycines are indicated in red, while glutamines and asparagines are in green. (B) Partial or full substitution of Q/N residues for G within the Sup35 nucleation domain resulted in the *ADE*^*+*^ degradation phenotype (*left*), and a step-wise increase in degradation rate (*right*). Partial substitution of G residues for Q/N residues within the A2 PrLD resulted in an *ade*^*-*^ phenotype (*left*), and corresponding decrease in degradation rate (*right*). Full substitution of remaining G residues for QN residues had mixed effects.

These results may also provide an explanation for Sup35’s resistance to degradation. Q/N residues were among the lowest scoring amino acids in the degradation libraries. The human PrLDs and the Sup35 ND differ most notably in their Q/N content; the Sup35 ND contains a much higher percentage of Q/N-residues, while the A1 and A2 core PrLDs are more G-rich. This suggests the simple hypothesis that the high Q/N-content of the Sup35 ND may protect highly aggregation-prone features from recognition by components of the proteostasis machinery. To test this hypothesis, two of the degradation-prone members of the A2 library and their Sup35 counterparts were chosen as initial substrates for mutagenesis. To examine the relationship between Q/N content and degradation, we mutated some or all of the Q/N’s in the Sup35 nucleation domain to G’s (Fig 10A). Similarly, we mutated some or all of the G’s in the A2 PrLDs to Q/N.

The rate of degradation of Sup35 correlated with Q/N-content in a dose-dependent manner. Partial substitution of Q/N-residues for G’s significantly increased the turnover rate of each Sup35 derivative and resulted in the emergence of the *ADE*^+^ phenotype (Fig 10B; S5 Fig). Substitution of the remaining Q/N’s for G’s further enhanced the rate of Sup35 degradation. Partial or full substitution of G’s for Q/N’s in the A2 PrLD resulted in a modest, albeit statistically significant increase in stability for one of the two mutagenized PrLDs. However, no stabilizing effect was observed for the second mutagenized PrLD, suggesting that other sequence features of the A2 PrLD besides Q/N content must contribute to its sensitivity to degradation. Therefore, in addition to their role in prion formation, Q/N residues help the Sup35 prion domain resist degradation by intracellular anti-aggregation systems.

## Discussion

Protein misfolding is a selective challenge faced by all cellular life. Misfolded proteins can result in proteotoxicity, either through loss-of-function of the native protein or through a toxic gain-of-function of the misfolded species. To address these selective challenges, eukaryotic cells possess extensive proteostasis machinery, which constitutively act to procure and maintain pools of natively folded proteins. The proteostasis machinery broadly consists of three main systems: 1) the protein chaperone network, which aids in nascent protein folding as well as the re-folding of partially or fully denatured proteins, 2) the ubiquitin-proteasome system, and 3) the autophagy system, which together aid in the destruction of aged, terminally misfolded, or aggregated proteins (for review, see [60, 61]).

Despite the constant surveillance of protein quality control systems, numerous diseases result from misfolding and aggregation of proteins. Additionally, a variety of proteins form functional aggregates that are involved in the regulation of various cellular processes [62–64]. Therefore, understanding how the proteostasis machinery detects misfolded proteins, and how some aggregation-prone proteins evade this detection, may provide insight into both functional and pathogenic aggregation.

One way through which proteostasis network components achieve specificity for misfolded proteins is by recognizing patches of solvent-exposed hydrophobicity [65–73]. Hydrophobic patches are generally buried in the interior of folded proteins [74], so exposed hydrophobicity can act as a signal of protein misfolding. Additionally, there is a strong correlation between hydrophobicity and aggregation propensity [75], so recognizing exposed hydrophobicity would seem to be an effective mechanism to recognize aggregation-prone misfolded proteins.

One well-characterized example that uses this mechanism is the yeast E3 ubiquitin ligase San1, a nuclear protein involved in the ubiquitin-proteasome degradation system [76]. San1 is a largely disordered protein that is particularly adept at targeting toxic misfolded proteins for degradation [77], primarily by recognizing exposed hydrophobic residues in substrates [58]. Interestingly, San1 recognition of these substrates tends to correlate with their insolubility, demonstrating the effectiveness of targeting hydrophobicity to prevent protein aggregation [57]. It should be noted that degradation of the A1- and A2-Sup35 fusions was independent of San1 (S6 Fig), so additional work will be required to identify the cellular factors responsible for recognition and degradation of these PrLDs. Identifying these factors and studying the mechanisms by which they recognize aggregation-prone proteins may help explain mechanistically how aromatic amino acids can promote aggregation without triggering PQC degradation.

Although our data is generally consistent with the idea that exposed hydrophobic amino acids promote recognition by the proteostasis machinery, our results provide some additional unexpected insights. First, in contrast to what has been proposed for San1, we show that aggregation propensity and recognition by the proteostasis machinery can be uncoupled in a composition-dependent manner: aromatic amino acids within the G-rich hnRNP PrLDs increase aggregation propensity without substantially enhancing recognition by the proteostasis machinery. Second, the ability of the proteostasis machinery to recognize hydrophobic patches was highly context dependent: the Q/N-rich Sup35 ND had the inherent capacity to mask otherwise degradation-promoting amino acids. These results highlight an important point related to the proteostasis of prion and prion-like domains. While it is sometimes useful to broadly categorize certain amino acids as “aggregation-promoting” or “degradation-promoting”, the effects of these amino acids may vary from protein to protein depending on the larger sequence context within which they are found, and on the interactions between these domains and cellular proteostasis factors. Since both the short sequence features and the surrounding context play such important roles in aggregation and degradation, future examination of the extent to which these heuristics apply to other aggregation-prone proteins and in other organisms would be interesting.

While PolyQ regions reportedly resist degradation by the proteasome [78] (although this too remains quite controversial [79, 80]; for review, see also [81, 82]), Sup35 has a roughly average half-life *in vivo* [83] suggesting that it is not inherently unusually resistant to degradation. Some evidence indicates that certain fragments of the Q/N-rich Sup35 prion domain exhibit a high rate of turnover [84], and the Sup35 prion domain can be proteolytically cleaved [85], indicating that the degradation and proteolytic systems are not incapable of processing the Sup35 prion domain *in vivo*. However, our results illuminate a principle fundamentally distinct from inherent stability–namely, sequences capable of potently inducing degradation in the G-rich PrLDs are, in some way, protected from the proteostasis machinery by surrounding Q/N-rich regions. Therefore, Q/N residues may potentiate the aggregation of prion domains, in part, by protecting aggregation-prone features from the proteostasis machinery. Although increased degradation and increased aggregation are not necessarily alternatives [84, 86], our results suggest that the composition of the Sup35 prion domain allows it to resist degradation, while maintaining the ability to form prions.

While the exact mechanism by which Sup35 resists degradation is unclear, high Q/N-content appears to play an important role. The Sup35 prion domain and the A1/A2 PrLDs are each predicted to be intrinsically disordered. However, the Sup35 prion domain is thought to form a collapsed but disordered structure [87], which may hide hydrophobic patches from the proteostasis machinery. High Q/N content may help mask hydrophobic patches by promoting a collapsed but disordered structure, or by shielding hydrophobic amino acids within these structures. Alternatively, rather than preventing the initial recognition of hydrophobic patches by the proteostasis machinery, Q/N residues may inhibit a downstream step in the subsequent events leading to degradation. Interestingly, the Q/N content of Sup35 is relatively well-conserved across independent *Saccharomyces cerevisiae* strains and between different yeast species [88, 89]. Although high Q/N content within the N-domain may be maintained by selection for multiple reasons, it is possible that the stabilizing effects of Q/N at least contribute to the observed compositional conservation.

Other features of the Sup35 prion domain besides Q/N content also seem well-suited to avoid detection by the degradation machinery, potentially explaining why increasing the Q/N content of the A2 PrLD was not sufficient to fully stabilize the PrLD. We previously showed that six amino acids are highly prion-promoting: F, Y, W, I, V, and M [42]. The Sup35 prion domain contains 23 of these highly prion-promoting amino acids, yet all except the initiating methionine are aromatic. Additionally, the prion-promoting amino acids are well-dispersed. There is only one position where two occur adjacent to each other, and almost all have adjacent Q/N residues. Thus, the Sup35 prion domain possesses many features that promote aggregation, yet avoids multiple features that can contribute to degradation. Furthermore, these biases are not unique to the Sup35 prion domain; most other yeast prion domains are also Q/N-rich, and tend to favor aromatic amino acids over non-aromatic prion-promoting amino acids [44].

Numerous labs have made extensive progress in defining how the amino acid sequence of a protein affects its intrinsic aggregation propensity. However, our results highlight that intrinsic aggregation propensity is only a small piece of the puzzle. A more complete understanding of functional and pathogenic protein aggregation requires a clearer view of how amino acid sequence affects interactions with other cellular proteins. Our results provide one unexpected piece to this puzzle, demonstrating that specific sequence features can promote protein aggregation, while simultaneously hiding from the proteostasis machinery.

## Materials and methods

### Strains and media

Standard yeast media and methods were used as previously described [90], except that YPD plates contained 0.5% yeast extract rather than the standard 1%. YPAD for all experiments contained the standard 1% yeast extract, as well as 0.02% adenine hemisulfate. Prion curing assays were performed for individual *ADE*^+^ isolates by streaking onto YPD with and without 4mM GuHCl, then re-streaking to YPD to test for loss of the *ADE*^+^ phenotype. In all experiments, yeast were grown at 30°C. The yeast strains used in this study were YER826/pER589 (α *kar1-1 SUQ5 ade2-1 his3 leu2 trp1 ura3 sup35*::KanMx) and YER1161 (α *kar1-1 SUQ5 ade2-1 his3 leu2 trp1 ura3 sup35*::KanMx *pdr5*::*HIS3*). pER589 expresses a truncated version of Sup35 lacking the prion domain (Sup35MC) as the sole copy of Sup35 in the cell. This plasmid was subsequently replaced by plasmid shuffling in order to assay activity of the full-length, randomly mutagenized hnRNP-Sup35 fusions.

### Generating mutant libraries

The A1-Sup35 and A2-Sup35 fusion libraries were generated in a manner similar to MacLea *et al*. [44]. Briefly, the N-terminal end and C-terminal end of each gene were amplified from a plasmid containing either the A1-Sup35 fusion or the A2-Sup35 fusion (pER595 for hnRNPA1 and pER697 for hnRNPA2; [37]). Oligonucleotides (from Integrated DNA Technologies) were used to re-amplify the respective products and incorporate a 24-nucleotide degenerate region in which each of the four nucleotides has a 25% probability of occurring at the first two positions of each codon, while C, G, and T each have a 33% probability of occurring at the final position of each codon. The N-terminal and mutagenized C-terminal products, which contain complementary segments, were mixed and re-amplified. The final PCR products were co-transformed with BamHI/HindIII-cut pJ526 into YER826 and plated on synthetic complete media lacking leucine (SC-Leu) to select for cells containing a recombined plasmid. Individual colonies were then picked and stamped onto media containing 5-Fluoroorotic acid (5-FOA) to select for loss of pER589.

### Determination of prion propensity scores and degradation propensity scores

After 5-FOA treatment, cells were transferred to YPAD, YPD, and SC-ade. After three days at 30°C, isolates for which more than 5 colonies appeared on SC-ade were identified and placed in a category (*ADE*^+^ library) separate from those with fewer than 5 colonies (*ade*^-^). Randomly selected representative isolates from both groups were sequenced to generate each library. The odds ratio for the *ADE*^+^ phenotype (*OR*_*A*_) for each amino acid was determined as follows:

$${OR}_{A}\;=\;\left[\frac{f_{D}}{1-f_{D}}\right]/\left[\frac{f_{N}}{1-f_{N}}\right]$$ (1)

where *f*_*D*_ represents the per residue frequency of the amino acid among the isolates that were able to grow on SC-ade, and *f*_*N*_ represents the per residue frequency of the amino acid among the naïve isolates (i.e., those that were unable to grow on SC-ade). Final degradation propensity scores for each amino acid (*DP*_*aa*_) were determined as follows:

$${DP}_{aa}\;=\;\text{ln}\;\left({OR}_{A}\right)$$ (2)

In addition, prion isolates were identified as previously described [42, 44] (Fig 1) and sequenced to generate the prion library. Briefly, the isolates that were initially unable to grow on SC-ade were pooled from the solid YPAD media and re-plated on SC-ade at approximately 10^6^ and 10^5^ cells per plate. After 3–5 days at 30°C, individual colonies were streaked onto YPD and YPD plus 4mM GuHCl to assay for prion loss. Odds ratios for prion activity (*OR*_*P*_) for each amino acid were determined as follows:

$${OR}_{P}\;=\;\left[\frac{f_{P}}{1-f_{P}}\right]/\left[\frac{f_{N}}{1-f_{N}}\right]$$ (3)

where *f*_*P*_ represents the per residue frequency of the amino acid among the prion-forming isolates. Final prion propensity scores (*PP*_*aa*_) were determined as follows:

$${PP}_{aa}\;=\;\text{ln}\;\left({OR}_{P}\right)$$ (4)

### Degradation assays

Cells were diluted to an optical density of 0.75 in liquid YPAD media and incubated with shaking at 30°C for 1hr before treatment with CHX, or DMSO for untreated cells. Where applicable, MG-132 was added to a final concentration of 10μg/mL 1 hr prior to addition of CHX. After the treatment period, the optical densities of all cultures were measured. 10mL of the least-dense culture for each strain were harvested. Based on the optical densities, the approximate number of cells harvested for each of the remaining cultures was normalized to the least-dense culture within each unique strain. Cells were pelleted by centrifugation at 3,000rpm for 5 minutes at 4°C. Cell pellets were lysed as previously described [57]. 30μL of prepared lysate were loaded onto a 12% polyacrylamide gel, transferred to a PVDF membrane, and probed with an appropriate antibody. Primary antibodies (all monoclonal) used in this study were: an anti-Sup35C (BE4 [91], kindly made available by Susan Liebman), an anti-GFP antibody (Santa Cruz Biotechnology), and an anti-FLAG antibody (Sigma). Blots were quantified using Image Studio Version 5.2. Background-subtracted intensities for all quantified blots can be found in S1 Table.

### Solubility assay

As with the degradation assays, yeast cultures were diluted to an optical density of 0.75 in liquid YPAD media and incubated with shaking at 30°C for 1hr before normalizing to the least-dense culture and harvesting. Cell pellets were re-suspended in 200μL of chilled non-denaturing lysis buffer (100mM TrisHCl, pH7.5, 200mM NaCl, 1mM EDTA, 5% Glycerol, 0.1% Triton-X 100, and Bond-Breaker TCEP solution (Thermo Fisher Scientific) and ProBlock Gold yeast protease inhibitor cocktail (Gold Biotechnology) to manufacturer recommendations; adapted from [92]), transferred to a round-bottom 2mL tube, and vortexed with a single large glass bead (~3mm diameter) on maximum speed for 10 minutes. Lysates were centrifuged gently (700 x g for 5 minutes at 4°C) to pellet unlysed cells and large cellular debris. 50μL of total lysate sample was mixed with 50μL of denaturing buffer (1% SDS, 8M urea, 10mM MOPS pH6.8, 10mM EDTA pH8.0, 0.01% bromophenol blue, and ProBlock Gold yeast protease inhibitor cocktail (Gold Biotechnology) to manufacturer recommendations). 100μL of remaining lysate was centrifuged at 16.3k rpm for 15 minutes at 4°C to pellet protein aggregates. The supernatant was removed and mixed 1:1 with denaturing buffer (soluble sample). The remaining pellet was resuspended in an equal volume of a 1:1 mixture of non-denaturing:denaturing buffer. Samples were boiled for 5 minutes, then centrifuged at 12,900 x g for 5 minutes before loading.

### Plasmid shuffling

Original strains were transformed with a covering plasmid expressing a version of Sup35 lacking the prion domain (Sup35MC) and a URA3 selectable marker. Transformants were passaged on SC-Ura until loss of the original plasmid expressing the A2-Sup35 fusion. After loss of the plasmid, strains were re-transformed with the original A2-Sup35 fusion plasmid and the URA3 covering plasmid counter-selected on 5-fluoroorotic acid (FOA). Color phenotypes for each strain were compared by streaking onto YPD.

## Supporting information

S1 Fig Prion-independent inactivation of A2-Sup35. A covering plasmid expressing a copy of Sup35 lacking the prion domain was shuffled into *ADE*^+^ strains with the sequences MMIWGRLA (A), AVLLWTSG (B), MVMFFECA (C), or LFVIASSK (D) within the mutagenized region of the A2 PrLD. In all cases, the cells became *ade*^-^ upon loss of the plasmid, but re-gained the *ADE*^+^ phenotype when the plasmid was re-introduced.
(TIF)

S2 Fig *ADE*^*+*^ strains do not contain detectable aggregates. WT Sup35 partitioned almost exclusively to the soluble (S) fraction in a known [*psi*^-^] strain, whereas a substantial proportion of Sup35 was found in the insoluble (I) fraction in a known [*PSI*^+^] strain. Fractionation of representative *ADE*^+^ isolates resulted in A2-Sup35 distributions consistent with the absence of large prion aggregates. Fractionation of a [*PRION*^+^] strain dependent upon the A2-Sup35 fusion resulted in a substantial portion of the A2-Sup35 protein in the insoluble fraction, consistent with A2-Sup35 forming prion aggregates.
(TIF)

S3 Fig Steady state protein levels for *ADE*^+^ and *ade*^-^ isolates are consistent with Ade assay thresholds. Steady state protein levels were determined in synthetic complete (SC) medium and synthetic complete medium lacking adenine (SC-ade) for all A2 PrLD (A) and Sup35 ND mutants (B) indicated in Fig 4. All protein levels are normalized to wild-type Sup35, which consistently had the highest steady state level. Data represent means ± SDs (n = 3). Protein levels for sequences that promoted the *ADE*^+^ phenotype when in the context of the A2 PrLD (MMIWGRLA, AVLLWTSG, MVMFFECA, and LFVIASSK) were grouped and statistically compared to grouped protein levels for sequences that promoted the *ade*^*-*^ phenotype (RVLDFSGF, WSCSQFRT, TPQTDQDW and SGNYNDFG). Since all mutant forms of the Sup35 ND significantly decreased steady state levels compared to wild-type Sup35, protein levels for wild-type Sup35 were excluded from statistical comparison. Groups were compared using a two-tailed Student’s t-test (p < 0.001, ***; p > 0.05, n.s.). The colors of the significance indicators correspond to the types of media indicated in the legends.
(TIF)

S4 Fig [*PIN*^+^] does not affect susceptibility or resistance to degradation. Wild-type and select hydrophobic insertion mutants for Sup35 and the A2-Sup35 fusion were expressed in a [*pin*^-^] strain (*Δrnq1*). (A,C) Insertion of as few as two hydrophobic residues within the A2 PrLD results in the appearance of the *ADE*^+^ phenotype and a corresponding increase in degradation rate. (B,D) Insertion of up to six hydrophobic residues within the Sup35 ND does not detectably increase degradation rate, and the cells are correspondingly unable to grow on SC-ade.
(TIF)

S5 Fig Degradation of the A2 PrLD and Sup35 ND depends on G/Q/N content. Protein levels for the A2 PrLD (A) and Sup35 ND (B) are indicated as a function of time after addition of CHX for western blots in Fig 10B. The 8-amino acid sequences substituted into the regions illustrated in Fig 10A are indicated above each graph. Data represent means ± SDs (n≥3). Protein levels for mutants that differ significantly from protein levels of the wild-type protein at the corresponding time point are indicated with a colored asterisk (two-tailed Student’s t-test; p < 0.05, *; p < 0.01, **; p < 0.001, ***).
(TIF)

S6 Fig Degradation of the A2 PrLD is San1-independent. Plasmids expressing A2-Sup35 fusions containing the indicated degradation-promoting sequences were shuffled into a *san1Δ* strain, and expressed as the sole copy of Sup35. Strains were plated on SC-ade, and YPD, and YPAD, and protein levels were assessed by western blot after treatment with CHX.
(TIF)

S1 Table Background-corrected intensity values for all quantified western blots. (XLSX)
