# Supplementary figures and images for: Sequence features governing aggregation or degradation of prion-like proteins
Source: PLoS Genet. 2018 Jul 13;14(7):e1007517. doi: 10.1371/journal.pgen.1007517 (PMC6059496; doi:10.1371/journal.pgen.1007517)

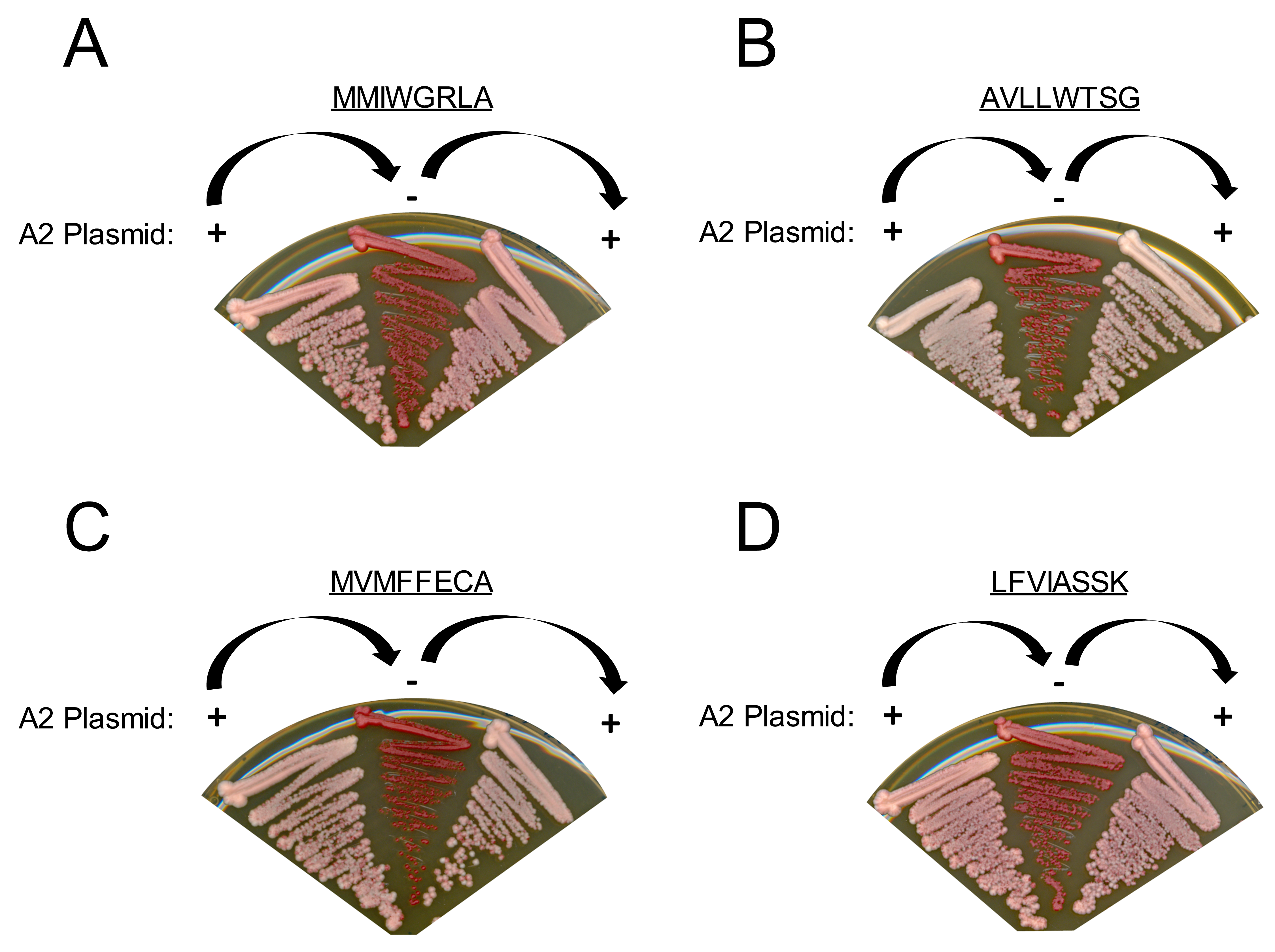

Supplement: S1 Fig — A covering plasmid expressing a copy of Sup35 lacking the prion domain was shuffled into ADE+ strains with the sequences MMIWGRLA (A), AVLLWTSG (B), MVMFFECA (C), or LFVIASSK (D) within the mutagenized region of the A2 PrLD. In all cases, the cells became ade- upon loss of the plasmid, but re-gained the ADE+ phenotype when the plasmid was re-introduced. (TIF) [file pgen.1007517.s001.tif]

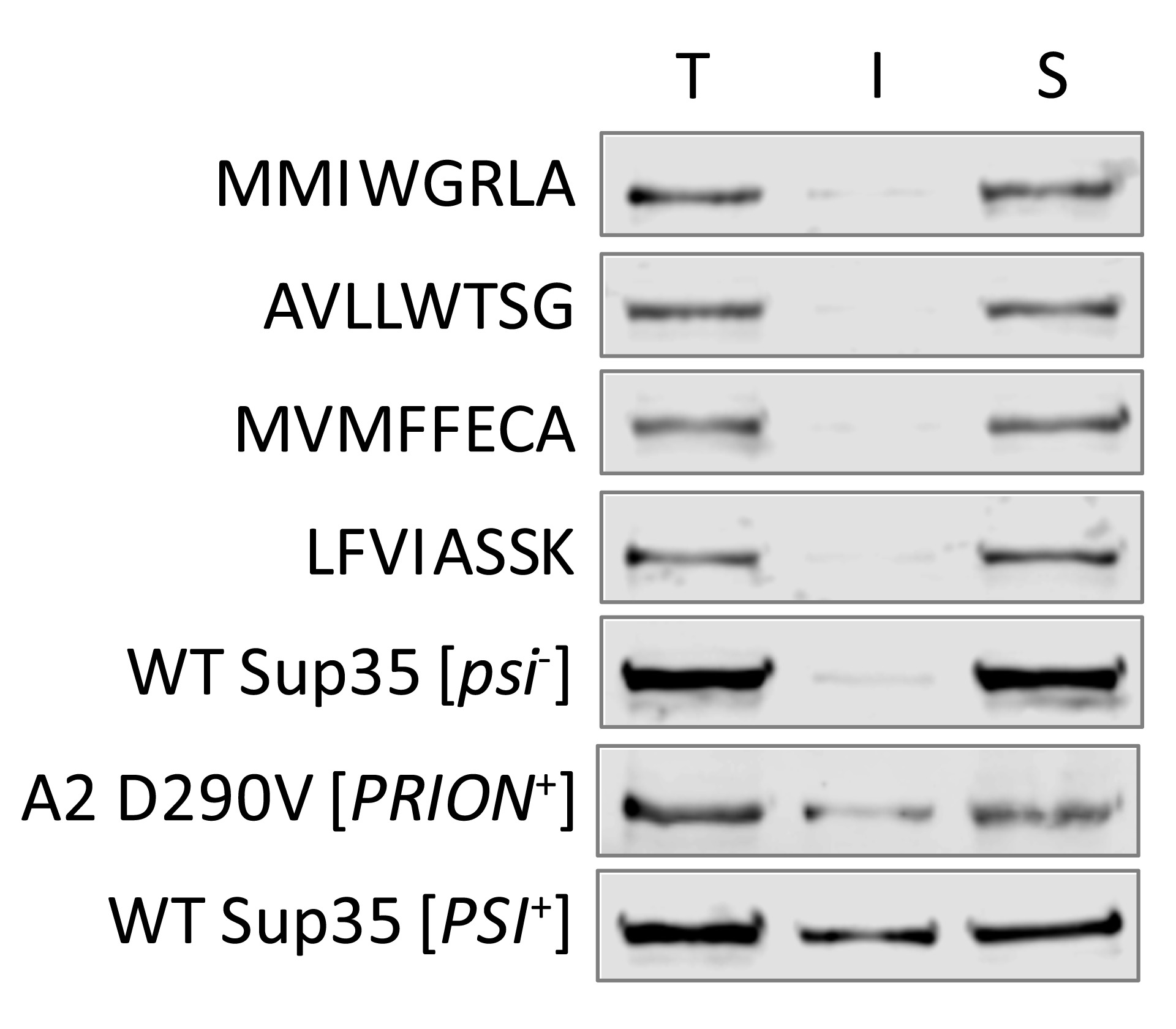

Supplement: S2 Fig — WT Sup35 partitioned almost exclusively to the soluble (S) fraction in a known [psi-] strain, whereas a substantial proportion of Sup35 was found in the insoluble (I) fraction in a known [PSI+] strain. Fractionation of representative ADE+ isolates resulted in A2-Sup35 distributions consistent with the absence of large prion aggregates. Fractionation of a [PRION+] strain dependent upon the A2-Sup35 fusion resulted in a substantial portion of the A2-Sup35 protein in the insoluble fraction, consistent with A2-Sup35 forming prion aggregates. (TIF) [file pgen.1007517.s002.tif]

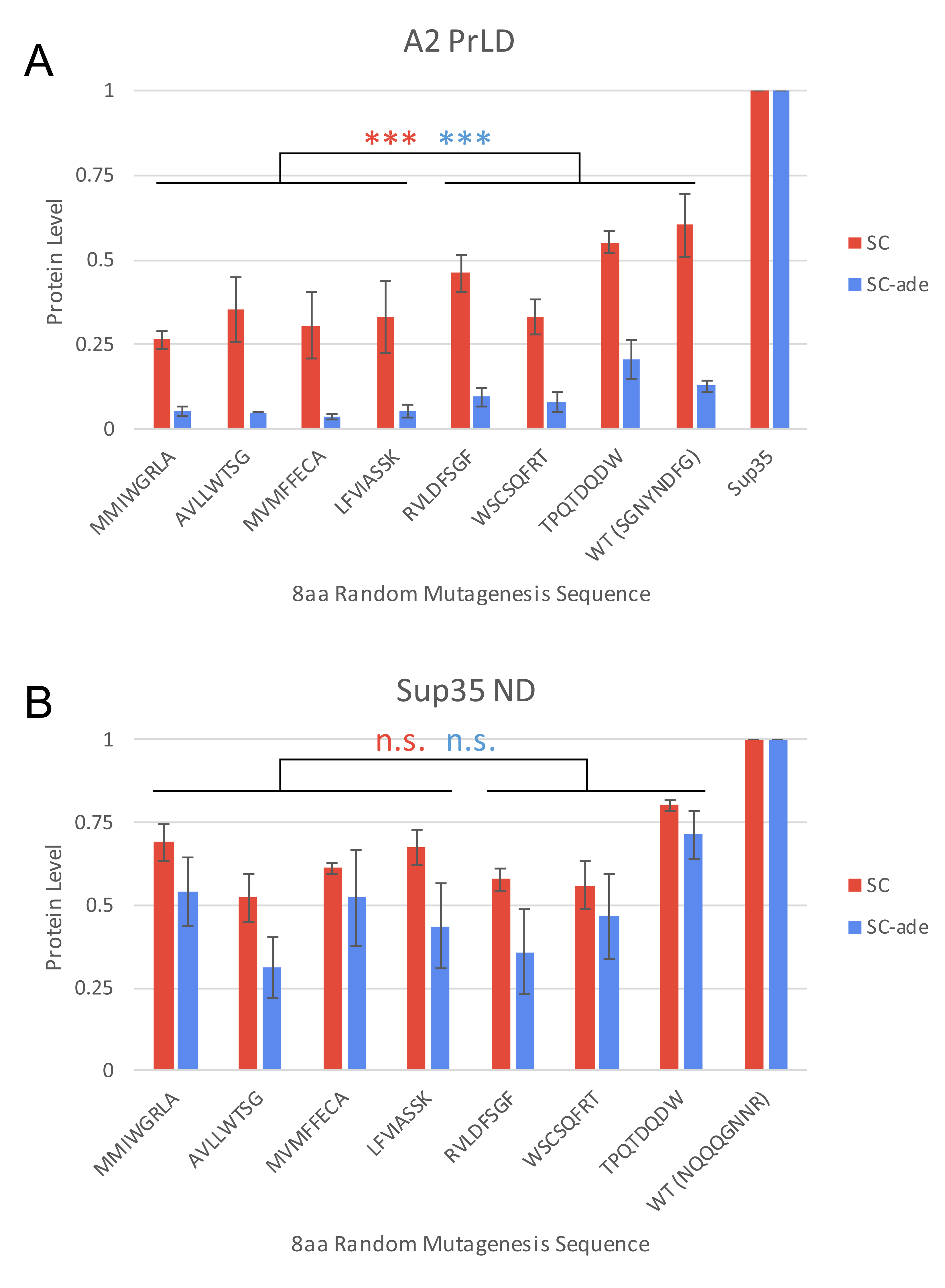

Supplement: S3 Fig — Steady state protein levels were determined in synthetic complete (SC) medium and synthetic complete medium lacking adenine (SC-ade) for all A2 PrLD (A) and Sup35 ND mutants (B) indicated in Fig 4. All protein levels are normalized to wild-type Sup35, which consistently had the highest steady state level. Data represent means ± SDs (n = 3). Protein levels for sequences that promoted the ADE+ phenotype when in the context of the A2 PrLD (MMIWGRLA, AVLLWTSG, MVMFFECA, and LFVIASSK) were grouped and statistically compared to grouped protein levels for sequences that promoted the ade- phenotype (RVLDFSGF, WSCSQFRT, TPQTDQDW and SGNYNDFG). Since all mutant forms of the Sup35 ND significantly decreased steady state levels compared to wild-type Sup35, protein levels for wild-type Sup35 were excluded from statistical comparison. Groups were compared using a two-tailed Student’s t-test (p < 0.001, ***; p > 0.05, n.s.). The colors of the significance indicators correspond to the types of media indicated in the legends. (TIF) [file pgen.1007517.s003.tif]

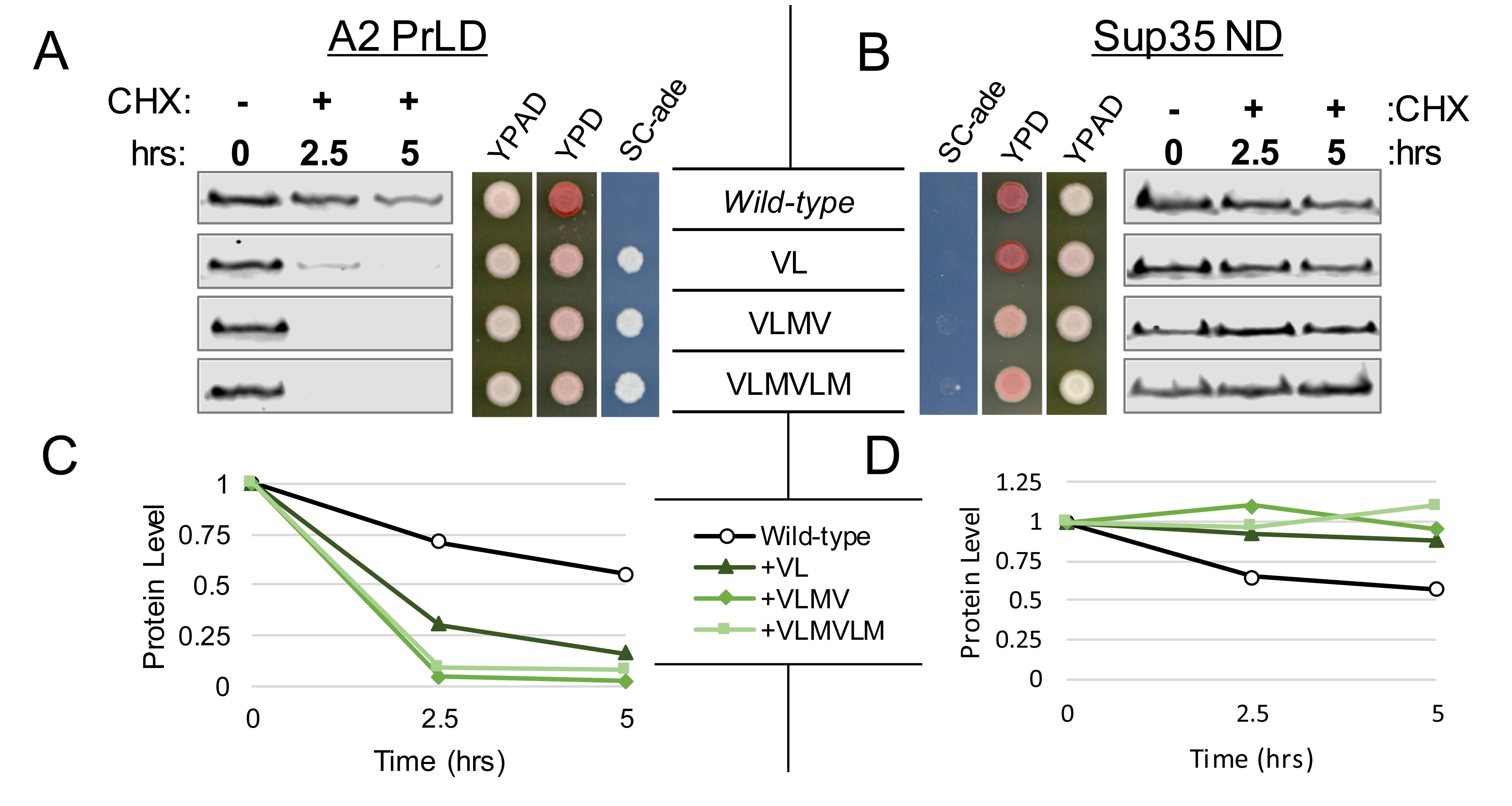

Supplement: S4 Fig — Wild-type and select hydrophobic insertion mutants for Sup35 and the A2-Sup35 fusion were expressed in a [pin-] strain (Δrnq1). (A,C) Insertion of as few as two hydrophobic residues within the A2 PrLD results in the appearance of the ADE+ phenotype and a corresponding increase in degradation rate. (B,D) Insertion of up to six hydrophobic residues within the Sup35 ND does not detectably increase degradation rate, and the cells are correspondingly unable to grow on SC-ade. (TIF) [file pgen.1007517.s004.tif]

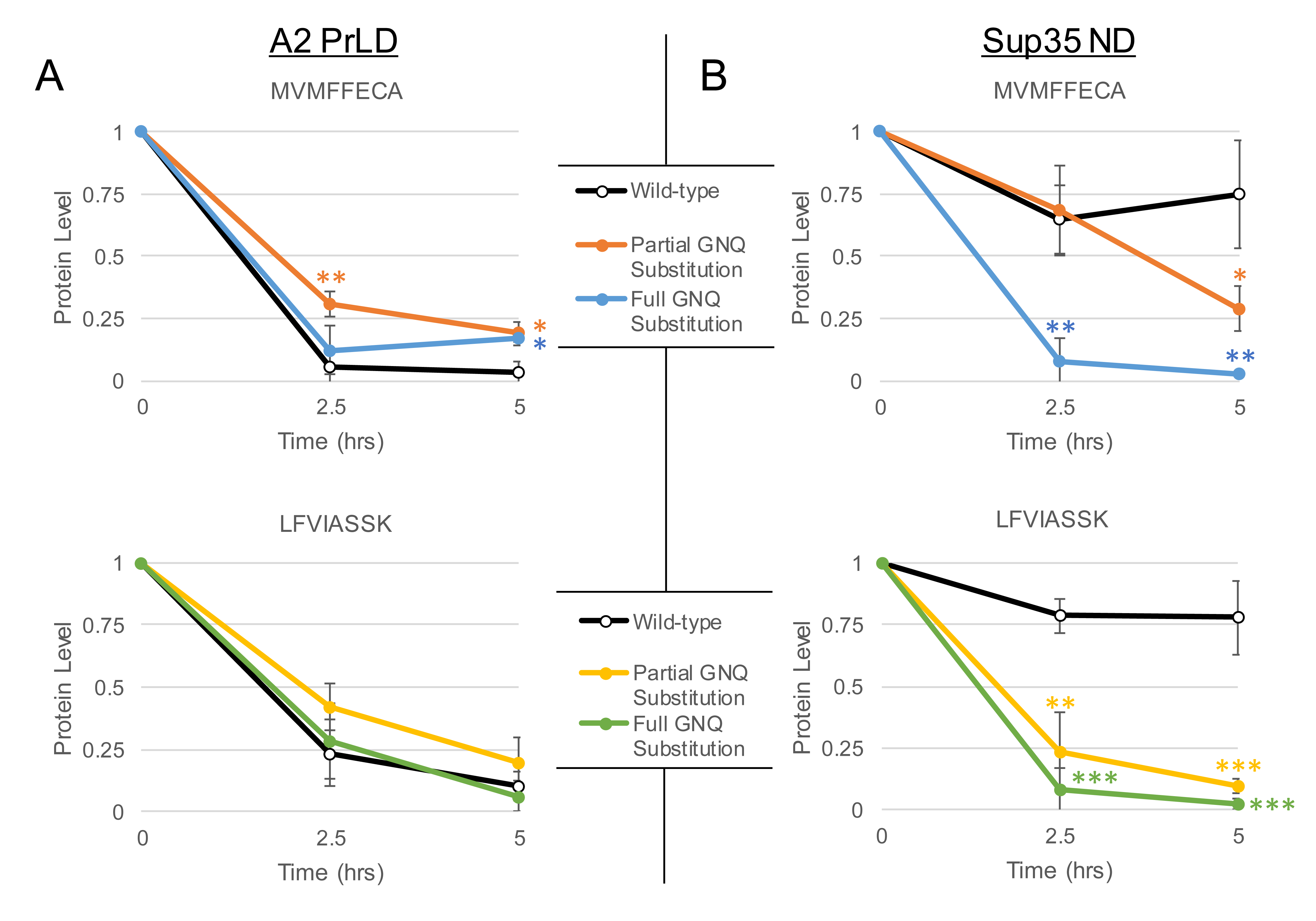

Supplement: S5 Fig — Protein levels for the A2 PrLD (A) and Sup35 ND (B) are indicated as a function of time after addition of CHX for western blots in Fig 10B. The 8-amino acid sequences substituted into the regions illustrated in Fig 10A are indicated above each graph. Data represent means ± SDs (n≥3). Protein levels for mutants that differ significantly from protein levels of the wild-type protein at the corresponding time point are indicated with a colored asterisk (two-tailed Student’s t-test; p < 0.05, *; p < 0.01, **; p < 0.001, ***). (TIF) [file pgen.1007517.s005.tif]

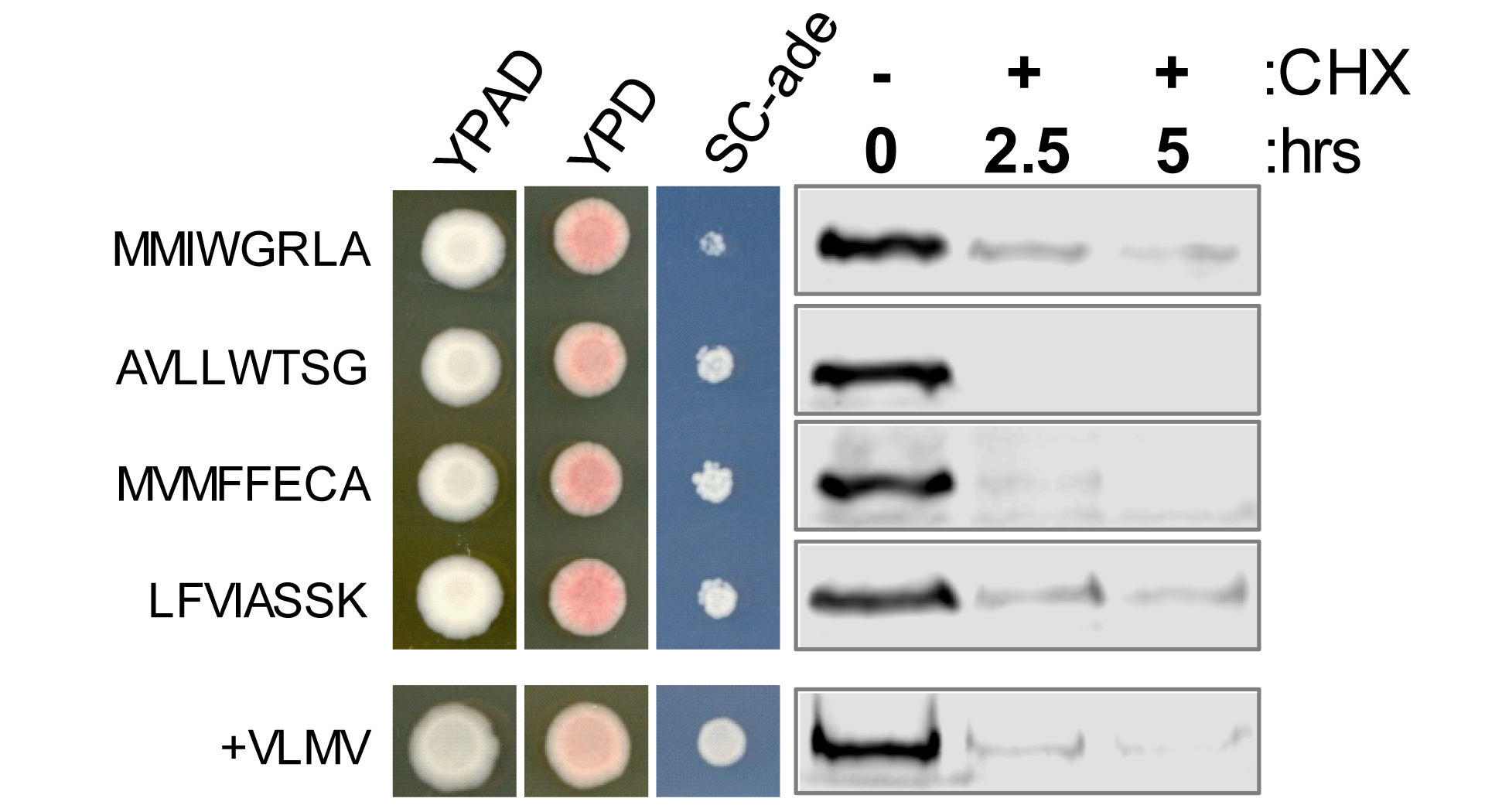

Supplement: S6 Fig — Plasmids expressing A2-Sup35 fusions containing the indicated degradation-promoting sequences were shuffled into a san1Δ strain, and expressed as the sole copy of Sup35. Strains were plated on SC-ade, and YPD, and YPAD, and protein levels were assessed by western blot after treatment with CHX. (TIF) [file pgen.1007517.s006.tif]
